# Divergence in chondrogenic potential between in vitro and in vivo of adipose- and synovial-stem cells from mouse and human

**DOI:** 10.1186/s13287-021-02485-5

**Published:** 2021-07-15

**Authors:** Ryota Chijimatsu, Satoshi Miwa, Gensuke Okamura, Junya Miyahara, Naohiro Tachibana, Hisatoshi Ishikura, Junya Higuchi, Yuji Maenohara, Shinsaku Tsuji, Shin Sameshima, Kentaro Takagi, Keiu Nakazato, Kohei Kawaguchi, Ryota Yamagami, Hiroshi Inui, Shuji Taketomi, Sakae Tanaka, Taku Saito

**Affiliations:** 1 Bone and Cartilage Regenerative Medicine, Graduate School of Medicine, The University of Tokyo, Tokyo, Japan; 2 Sensory and Motor System Medicine, Graduate School of Medicine, The University of Tokyo, Tokyo, Japan; 3 Orthopaedic Surgery, Osaka Rosai Hospital, Osaka, Japan; 4 Avenue Cell Clinic, Tokyo, Japan

**Keywords:** Chondrogenesis, Stem cell transplantation,, Transforming growth factor β (TGF-β), Adipose stem cells, Synovial stem cells, Somatic stem cells

## Abstract

**Background:**

Somatic stem cell transplantation has been performed for cartilage injury, but the reparative mechanisms are still conflicting. The chondrogenic potential of stem cells are thought as promising features for cartilage therapy; however, the correlation between their potential for chondrogenesis in vitro and in vivo remains undefined. The purpose of this study was to investigate the intrinsic chondrogenic condition depends on cell types and explore an indicator to select useful stem cells for cartilage regeneration.

**Methods:**

The chondrogenic potential of two different stem cell types derived from adipose tissue (ASCs) and synovium (SSCs) of mice and humans was assessed using bone morphogenic protein-2 (BMP2) and transforming growth factor-β1 (TGFβ1). Their in vivo chondrogenic potential was validated through transplantation into a mouse osteochondral defect model.

**Results:**

All cell types showed apparent chondrogenesis under the combination of BMP2 and TGFβ1 in vitro, as assessed by the formation of proteoglycan- and type 2 collagen (COL2)-rich tissues. However, our results vastly differed with those observed following single stimulation among species and cell types; apparent chondrogenesis of mouse SSCs was observed with supplementation of BMP2 or TGFβ1, whereas chondrogenesis of mouse ASCs and human SSCs was observed with supplementation of BMP2 not TGFβ1. Human ASCs showed no obvious chondrogenesis following single stimulation. Mouse SSCs showed the formation of hyaline-like cartilage which had less fibrous components (COL1/3) with supplementation of TGFβ1. However, human cells developed COL1/3+ tissues with all treatments. Transcriptomic analysis for TGFβ receptors and ligands of cells prior to chondrogenic induction did not indicate their distinct reactivity to the TGFβ1 or BMP2. In the transplanted site in vivo, mouse SSCs formed hyaline-like cartilage (proteoglycan+/COL2+/COL1−/COL3−) but other cell types mainly formed COL1/3-positive fibrous tissues in line with in vitro reactivity to TGFβ1.

**Conclusion:**

Optimal chondrogenic factors driving chondrogenesis from somatic stem cells are intrinsically distinct among cell types and species. Among them, the response to TGFβ1 may possibly represent the fate of stem cells when locally transplanted into cartilage defects.

**Supplementary Information:**

The online version contains supplementary material available at 10.1186/s13287-021-02485-5.

## Introduction

It is widely accepted that local injury of articular cartilage associated with joint trauma does not regenerate spontaneously. Transplantation of mesenchymal stem cells (MSCs) derived from bone marrow, synovium, and adipose tissue has shown to be a promising strategy for cartilage regeneration. Several clinical trials using MSCs have been completed or are ongoing [1, 2], although the actual outcome of transplantation of MSCs remain unclear.

There are conflicting reports on the reparative mechanisms of local transplantation of MSCs. Koga et al. reported that transplantation of bone marrow MSCs (BMSCs) or synovial MSCs (SMSCs) repaired cartilage defects with differentiation of transplanted cells to chondrocytes in an in vivo environment in a rabbit osteochondral defect model [3]. After transplantation, the cells were engrafted and functioned as chondrocytes/cartilage over 6 months [4]. On the other hand, Nakamura et al. found in a porcine study that the transplanted SMSCs repaired the cartilage defect without differentiation to chondrocytes; moreover, the transplanted SMSCs disappeared within a month [5]. Recently, many studies have highlighted that the signaling effects of transplanted stem cells lead to endogenous repair by host stem cells through the secretion of growth factors, cytokines, microRNAs, extracellular vesicles, and/or cell-cell contact [6, 7]. Thus, distinct mechanisms may be involved in transplantation therapy.

Several studies have suggested a relationship between in vitro chondrogenic potential and in vivo cartilage regeneration [3, 8, 9]. Among MSCs, SMSCs are reported to provide superior chondrogenic potential in humans [10, 11], rodents [12, 13], rabbit [3], pigs [5], and dogs [14]. Based on those findings, clinical trials of transplantation of SMSCs for articular cartilage defects have been recently reported [15, 16]. However, Dickhut et al. showed that transforming growth factor-β3 (TGFβ3) completely induced chondrogenesis from BMSCs, which was evident with the formation of tissue rich in type 2 collagen (COL2) and proteoglycan in vitro, whereas the stimulation with TGFβs alone was insufficient for SMSCs and adipose MSCs (AMSCs). The formed tissues contained a low amount of glycosaminoglycans and COL2 [17, 18]. Previous studies have shown that supplementation with bone morphogenic proteins (BMPs) is necessary for induction of chondrogenesis from SMSCs and AMSCs [17, 19–21]. Moreover, other supplements such as serum and glucocorticoids also affect the chondrogenesis [19, 22–26]. Thus, conditions for adequate chondrogenesis are intrinsically different among MSCs, implying that chondrogenic potential is probably miscalculated depending on assay conditions.

For further understanding the mechanisms underlying the action of stem cells transplantation for cartilage repair therapy, we aimed to evaluate the in vitro and in vivo chondrogenic potential of two different somatic stem cell types derived from synovium and adipose tissue in mice and humans, respectively.

## Materials and methods

### Isolation and culture of somatic stem cells

Mouse somatic stem cells were isolated from 8-to-10-week-old C57BL/6 or CAG-EGFP C57BL/6. Mouse synovial stem cells (mSSCs) were established from knee infrapatellar fat pad as previously reported with slight modifications [13, 27]. In brief, synovium containing infrapatellar fat pad was surgically dissected and incubated in 10% fetal bovine serum (FBS; Sigma-Aldrich, MO, USA)-DMEM (Nacalai Tesque (Nacalai), Kyoto, Japan) containing 500 U/mL collagenase type 1 (Worthington, NJ, USA) at 37 °C with gentle rotation. After 1 h, the digested tissues were passed through a 70-μm strainer and washed twice. The isolated cells were cultured with growth media (10% FBS-DMEM supplemented with 1 ng/mL bFGF (FUJIFILM Wako Pure Chemical Corporation (Wako), Osaka, Japan) at 37 °C in a humidified atmosphere with 5% CO_2_ and 3% O_2_. Mouse adipose stem cells (mASCs) were obtained from the stromal vascular fraction of the inguinal fat tissue by collagenase digestion for 30 min. Only dissociated cells were collected using a 70-μm strainer and cultured with growth media at 37 °C in a humidified atmosphere with 5% CO_2_ and 3% O_2_. Cultured cells were passaged with 0.25% trypsin/ethylenediaminetetraacetic acid (EDTA) at 80% confluency and replated at a density of 5000 cells/cm^2^. The medium was changed three times per week. These cells have been already characterized in our previous report [27]. The cells were used for further experiments after 12–14 days of culture, as mouse somatic stem cells are sensitive to senescence [27, 28]. We prepared over three batches obtained from several mice with mixed gender at different timings.

Human subcutaneous adipose tissue and synovium were obtained from osteoarthritis (OA) patients (*N* = 6) during total knee arthroplasty (TKA) in accordance with a protocol approved by the institutional ethics committee. Written informed consent was obtained from all patients. The age, gender, and Kellgren-Lawrence grade of the patients are listed in Additional file 1. Adipose tissues were resected from the incision site at the knee, and synovium was resected from the suprapatellar pouch. Human ASCs (hASCs) were isolated as mentioned above and human SSCs (hSSCs) were isolated as previously established [18]. Human cells were cultured with growth media at 37 °C in a humidified atmosphere with 5% CO_2_. Those cells were passaged by treatment with 0.25% trypsin/EDTA at 80% confluency (approximately once per week) and replated at a density of 5000 cells/cm^2^. The media was changed twice per week. Human cells at passage 3 were used for further experiments.

### Chondrogenic induction

To obtain cell pellets, 2 × 10^5^ cells were centrifuged in polypropylene tube and cultured in growth medium. The next day, the medium was exchanged to chondrogenic medium (DMEM, 1% ITS+Premix (Corning, NY, USA), 50 μg/mL L-ascorbic acid 2-phosphate (Sigma-Aldrich), 40 μg/mL L-proline (Sigma-Aldrich)) supplemented with 100 ng/mL BMP2 (Medtronic, Dublin, Ireland), and 10 ng/mL TGFβ1 (ORIENTAL YEAST, Tokyo, Japan). Dexamethasone (Sigma-Aldrich) was used with TGFβ1 at concentration of 10 nM [19]. The cell pellets were maintained with 0.5 mL medium at 37 °C in a humidified atmosphere with 5% CO_2_. The medium was replaced twice per week.

### Animal experiment

Twelve-week-old male mice were anesthetized by an intraperitoneal injection of a mixture of 0.3 mg/kg of medetomidine, 4.0 mg/kg of midazolam, and 5.0 mg/kg of butorphanol. For both knees, the femoral trochlear grooves were exposed via a medial parapatellar incision with laterally patellar dislocation. An osteochondral defect (diameter, 0.5 mm; depth, approximately 0.5 mm) was created in both knees by manual drilling. A pellet of 5 × 10^4^ cells prepared by overnight culture with growth media were transplanted into the defects, and then patellar dislocation was reduced. The joint capsule and the skin were sutured as separate layers. After surgery, mice were allowed to be active without any fixation device or immobilization. C57BL/6 and CAG-EGFP C57BL/6 mice were used for allograft study and C.B-17 SCID mice were used for xenograft study.

### Micro-computed tomography

Whole knee joints were scanned using micro-computed tomography (μCT; inspeXio SMX-100CT system (Shimadzu, Kyoto, Japan)) at a resolution of 12 μm per voxel using the following consistent parameters: 75 kV and 140 mA. Three-dimensional images of bone were analyzed using the TRI/3D-BON software (RATOC System Engineering, Tokyo, Japan). Bone volume (BV) and bone mineral density (BMD) at the area of osteochondral defect trimmed into a cylinder (diameter, 0.5 mm; depth, 0.4 mm) were calculated as described previously [29].

### Histology and histochemistry

The samples were fixed in 10% neutral buffered formalin and embedded in paraffin wax, which was followed by serial dehydration using ethanol and clearance using xylene. For bone tissues, samples were decalcified with 10% EDTA (pH 7.4) after formalin fixation and delipidation using ethanol. Sections cut into 4-μm thickness were used for Safranin-O/fast green/hematoxylin staining (Saf-O), Alcian blue (pH 1.0) staining (AB), tartrate-resistant acid phosphatase (TRAP) staining, and immunohistochemistry. The information regarding the antibodies and reaction conditions is listed in Additional file 2. After reaction with HPR or AP conjugated antibodies, positive signal color was developed with Histofine Simple stain DAB (NICHIREI BIOSCIENCES, Tokyo, Japan), Vina Green Chromogen Kit (BIOCARE MEDICAL, CA, USA), or ImmPACT Vector Red AP Substrate Kit (VECTOR LABORATORIES, CA, USA). Positively stained areas were measured using ImageJ (National Institutes of Health, MD, USA), ImageScope (Leica Microsystems, Wetzlar, Germany), and BZX-700 (Keyence, Osaka, Japan).

### mRNA expression analysis

Cell pellets were initially homogenized with zirconia beads in TRI Reagent (Cosmo Bio, Tokyo, Japan). Total RNA was then extracted using Direct-zol RNA Kit (Zymo Research, CA, USA) according to the manufacturer’s protocol. Total RNA was reverse transcribed to cDNA using ReverTra Ace qPCR RT Master Mix (TOYOBO, Osaka, Japan). Quantitative reverse transcription polymerase chain reaction was performed using THUNDERBIRD SYBR qPCR Mix (TOYOBO) and Thermal Cycler Dice Real Time System III (TaKaRa Bio, Shiga, Japan). The information about primers is listed in Additional file 3. The expression levels normalized to the levels of glyceraldehyde 3-phosphate dehydrogenase (GAPDH) were measured using absolute standard curve method [30] or delta CT method [31].

### Transcriptome analysis

Total RNA extracted from human cells was used for transcriptome sequencing. The sequence libraries were prepared using a NEBNext Ultra II RNA Library Prep Kit for Illumina (New England Biolabs, MA, USA) according to the manufacturer’s protocol. Sequencing was performed using an Illumina HiSeq 4000 System with 2 × 150 bp paired-end reads (Veritas Genetics, MA, USA). The raw sequence data were filtered to remove adaptor sequences, low-quality reads, sequences with a high content of N, and reads < 50 bp length by using Trimmomatic (ver.0.39). The filtered data were aligned against the human reference genome (GRCh38.p13) using STAR (ver.2.7.3a). The gene expression counts and transcripts per million value (TPM) were calculated by RSEM (ver.1.3.3). Principal component analysis and hierarchical clustering was conducted by iDEP.91 (http://bioinformatics.sdstate.edu/idep/). For enrichment analysis in specific pathways, genes are collected from several gene sets related to TGFβ receptor signaling (M2642), proteoglycan (M15611, M12097, M13795, and M13500), and chondrogenesis/cartilage development (M10512, M14448, M11632, M34061, M15986, M10632, and M13025) from MSigDB v6.0. Heatmaps were created in R packages based on Log2(TPM + 1) or *Z* score of TPM values.

### Western blotting

Cells were lysed with T-PER (Thermo Fisher Scientific (Thermo), MA, USA) containing protease and phosphatase inhibitors (Nacalai). Following mixing with 4× LDS sample buffer (Thermo) and DTT (Nacalai), the samples were boiled at 70 °C for 10 min; 10 μg protein was applied to each lane of 4–12% Bolt Bis-Tris Plus precast polyacrylamide gel (Thermo) and separated by electrophoresis. For cell pellets, three replicates were pooled, and one-fifth volume of lysate was used for electrophoresis. Subsequently, the gels were transferred onto a PVDF membrane (Wako) using a Mini Blot Module (Thermo). After blocking with Blocking One (Nacalai) for 30 min, the membranes were probed with the following antibodies overnight at 4 °C or 1 h at room temperature: anti-phospho-Smad1/5 (#9516, CST, MA, USA), anti-Smad1 (#9743, CST), anti-phospho-Smad2 (#3108, CST), and anti-Smad2/3 (#8685, CST), anti-SOX9 (#82630, CST), HRP-conjugated-anti-GAPDH (#HRP-60004, Proteintech, IL, USA), HRP-conjugated-anti-rabbit IgG (#7074, CST). Then, immunoreaction was visualized with ChemiLumi One Super (Nacalai) and iBright 1000 (Thermo). The band signals were measured using iBright Analysis Software (Thermo).

### Statistical analysis

All data are presented as boxplot or bar plot with each value plotted as dot. Student’s unpaired *t* test was used to compare two groups, and one-way or two-way analysis of variance (ANOVA) with post hoc Tukey honestly significant difference test or Dunnet test was used for multiple groups. All statistical analysis was performed using SPSS software (IBM, Armonk, NY, USA, version 22.0). A *p* value less than 0.05 was considered statistically significant.

## Results

### TGFβ1 induced chondrogenesis from mSSCs but not mASCs in vitro

Previous reports indicate that optimal chondrogenic conditions differ among cell types [17, 32, 33]. To evaluate the actual chondrogenic capacity, in vitro pellet culture under stimulation with BMP2, TGFβ1, or their combination (B + T) was performed. As a reference, neither mASCs nor mSSCs differentiated into chondrocyte in the basal chondrogenic medium (see Additional file 4).

**Fig. 1:**
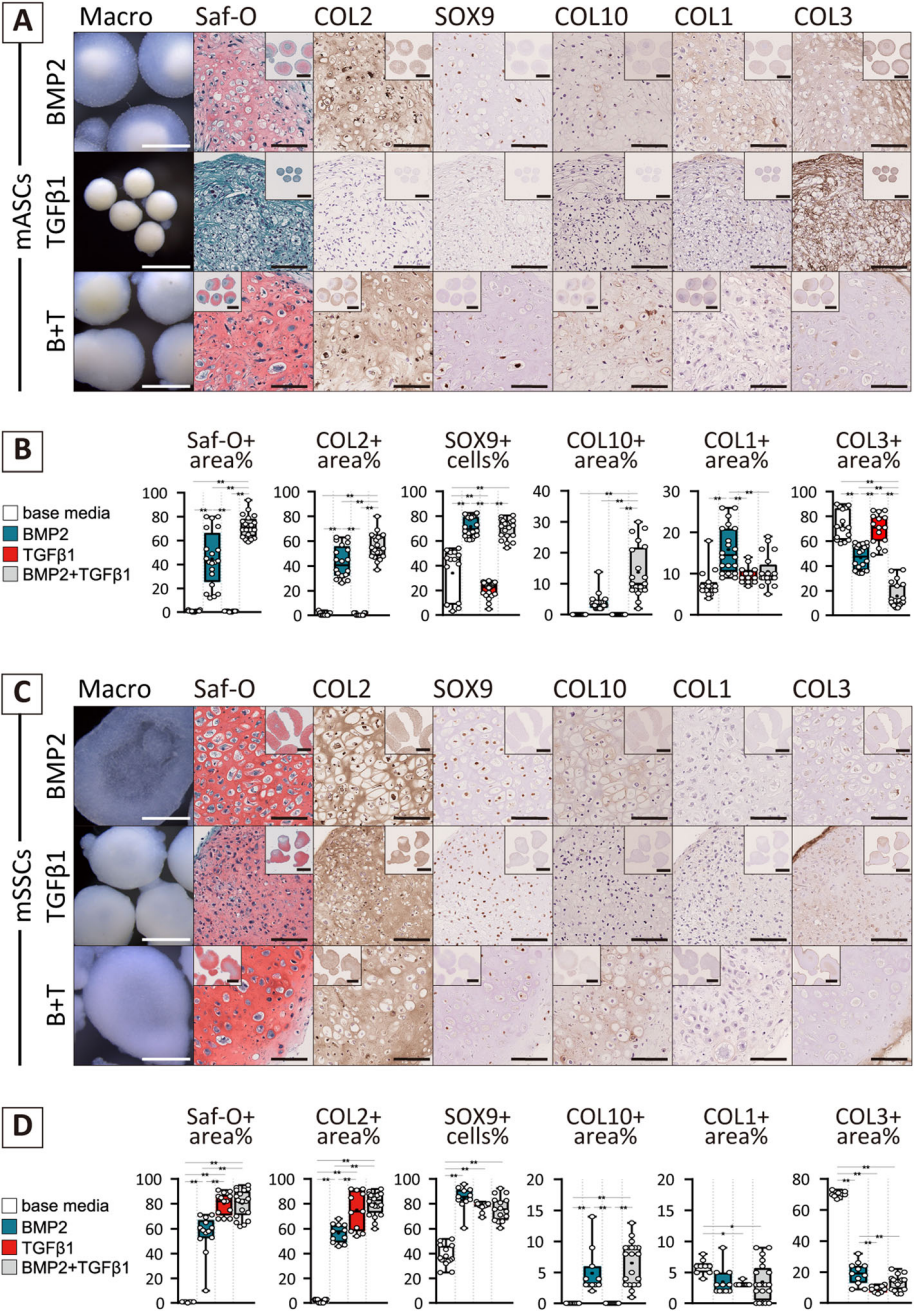
Chondrogenic potential of mASCs and mSSCs in the supplementation with BMP2 and/or TGFβ1. **A, C** Macroscopic view and histological images for chondrogenic pellet of cultured with BMP2 and/or TGFβ1 for 14 days. Representative data are shown (*N* = from over three lots). Scale bars = 1 mm (macro view and low-magnification histology) and 100 μm (high-magnification histology). **B, D** Quantification of IHC positive stained area expressed as a box plot with a dot plot. Significance was assessed using one-way ANOVA with post hoc Tukey honestly significant (HSD) difference test (*, *p* < 0.05; **, *p* < 0.01)

When mASC pellets were cultured under those three conditions, chondrogenic differentiation ascertained by semi-translucent tissue formation composed of proteoglycan and COL2 was recognized with supplementation of BMP2 (Fig. 1A, B). However, fibrotic matrices composed of type 1 and 3 collagen were observed in the chondrogenic tissues, indicating fibrocartilage development. Although there was no evidence for chondrogenesis in TGFβ1-treated mASCs even at the mRNA levels (see Additional file 5), TGFβ1 facilitated BMP2-induced chondrogenesis with reduction of COL1/3 component. In contrast, mSSCs underwent chondrogenesis under all conditions with the formation of a semi-translucent tissue with SOX9-positive chondrocytes embedded in the homogenous extracellular matrices enriched in proteoglycan, type 2 collagen, and low amount of COL1/3 (Fig. 1C, D). These observations show that mSSCs could form hyaline-like cartilaginous tissue. COL10, a hypertrophic marker, was observed in the area surrounding the hypertrophic cell lacuna only with BMP2 supplementation. The same trends were observed and validated at gene levels (see Additional file 5). Taken together, mASCs and mSSCs showed the potential for chondrogenesis but the suitable conditions were intrinsically different.

### mSSCs, but not mASCs, formed cartilaginous tissue in the osteochondral defect in vivo

**Fig. 2:**
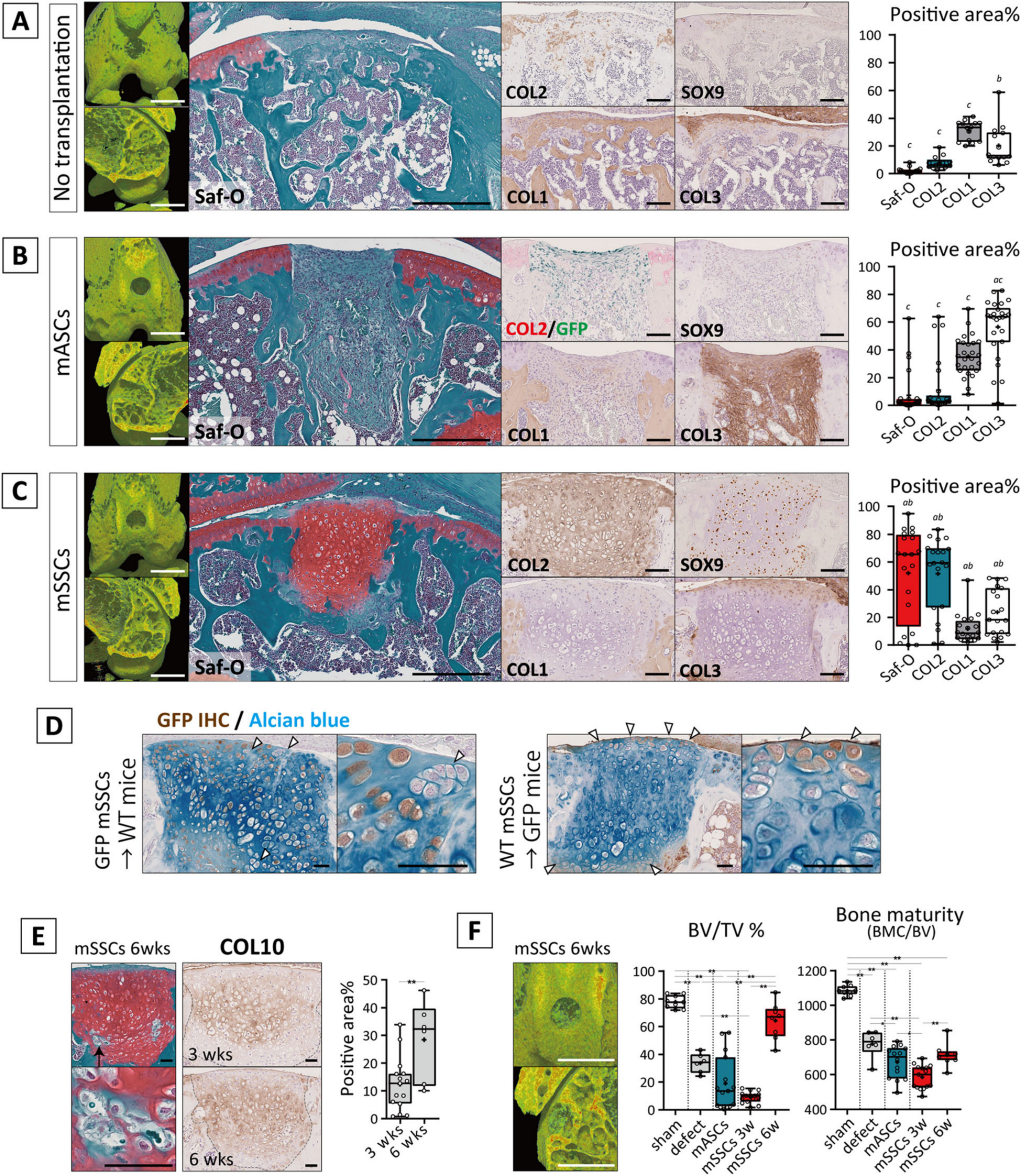
In vivo chondrogenic differentiation of mASCs and mSSCs. **A–C** Mouse osteochondral model of non-transplanted, mASCs-transplanted, and mSSCs-transplanted group at day 21. Micro CT and histological images with the qualification for their positive stained area in the region of approximately 0.5 mm width and 0.4 mm depth are shown. Scale bars = 1 mm (μCT), 500 μm (Saf-O), and 100 μm (IHC images). Significance was assessed using one-way ANOVA with post hoc Tukey HSD test (*a*, *p* < 0.05 compared with defect; *b*, *p* < 0.05 compared with mASCs; *c*, *p* < 0.05 compared with mSSCs). **D** IHC for GFP counterstained with Alcian blue. Arrow heads indicate host derived cells. Representative data are shown (*N* = 5 for the GFP-mSSC transplantation group and *N* = 4 for the WT-mSSC transplantation). Scale bars = 50 μm. **E** Representative images for Saf-O and IHC for COL10 of the specimens of mSSC-transplanted group at 6 weeks. Break line indicates the area for neo-cartilaginous tissue. Scale bars = 50 μm. Significance of COL10-positive area between 3 and 6 weeks was assessed using Student’s unpaired *t* test (**, *p* < 0.01). **F** Micro CT images and their analysis for bone volume of total volume (approximately 0.5 mm width and 0.4 mm depth) and bone maturity calculated according to bone mineral component in bone volume. Scale bars = 1 mm. Significance was assessed using one-way ANOVA with post hoc Tukey honestly significant (HSD) difference test (*, *p* < 0.05; **, *p* < 0.01)

The reparative capacities of mASCs and mSSCs in the osteochondral defect were evaluated by transplantation of the cell pellet into osteochondral defects. The osteochondral defect with no transplantation showed spontaneous repair of the subchondral bone, but the articular cartilage region did not regenerate and was covered with fibrous tissue (*N* = 11/14) or exposed bone (*N* = 3/14) (Fig. 2A). When mASCs were transplanted, the defects were filled with fibrous tissue with no regeneration of articular cartilage (Fig. 2B). Although, COL1 is a major fibrous tissue component, its detection was complicated for the assessment of fibrous tissue because the surrounding bone was also positive for COL1. Therefore, COL3, which is another fibrous component, was adapted in this study and strikingly signified the fibrotic area. GFP detection demonstrated that transplanted cells remained within the transplanted site without differentiation toward chondrocytes or osteoblasts, resulting in the impeded spontaneous repair of subchondral bone. In some cases, Saf-O^+^/COL2^+^ area was partially observed (*N* = 4/25); however, they were fibrous (*N* = 3/25) or ectopic site that protruded from the articular cartilage region (*N* = 1/25) (see Additional file 6).

On the other hand, when mSSCs were transplanted into the chondral defect, cartilaginous tissue rich in proteoglycans and COL2 but low in COL1/3 (*N* = 15/20) was newly generated (Fig. 2C). Interestingly, in all unrepaired cases (*N* = 5/20), defect sites were located in the distal femur groove and the host synovium tissue was infiltrated from enthesis of crucial ligaments to the defect site (see Additional file 7). GFP mice-derived mSSCs were detected at neo-cartilage tissue, indicating that mSSCs differentiated to chondrocytes and formed cartilage tissue in vivo (Fig. 2D). Notably, GFP-negative cells were also detected within the neo-cartilage tissue, particularly in the articular surface area and the boundary of bone marrow. To investigate the contribution of host cells, wild-type (WT)-derived mSSCs were transplanted into GFP mice. As a result, approximately 20% host cells were also incorporated for cartilage regeneration with differentiation toward chondrocytes (GFP-positive cell rate: 83.1 ± 6.6% (*N* = 5) in GFP cell transplantation to WT mice and 20.8 ± 11.4% (*N* = 4) in WT cell transplantation to GFP mice). At 6 weeks, remodeling of the neo-cartilaginous tissue was histologically observed in the deep zone (Fig. 2E). COL10 was observed from the middle to the deeper area of the neo-cartilage, which expanded at 6 weeks. The μCT analysis revealed that ossified volume of neo-tissues increased at 6 weeks but the mineral density did not mature like bone, suggesting the proceeding cartilage ossification (Fig. 2F). These findings indicate that mSSCs repaired the osteochondral defect via endochondral development and that accompanied with the stimulation of host cells to differentiate toward chondrocytes.

### TGFβ receptor derived-signaling is necessary for chondrogenesis

mASCs and mSSCs demonstrated chondrogenic potential in vitro but only mSSCs could differentiate to chondrocytes in vivo. Based on these results, it should be specified that the chondrogenic response to TGFβ1 was observed only in mSSCs in vitro. Phosphorylated Smad2/3 and Smad1/5/8 are the major downstream of TGFβ1 and BMP2 signaling, respectively [34]. In vivo specimens transplanted with mSSCs showed strong pSmad1 and weak pSmad2 activation during chondrogenesis. On the other hand, the fibrous area formed by mASCs contained cells weakly positive for pSmad1 and negative for pSmad2 (see Additional file 8). Therefore, the TGFβ-Smad2/3 pathway is important during in vivo chondrogenesis.

**Fig. 3:**
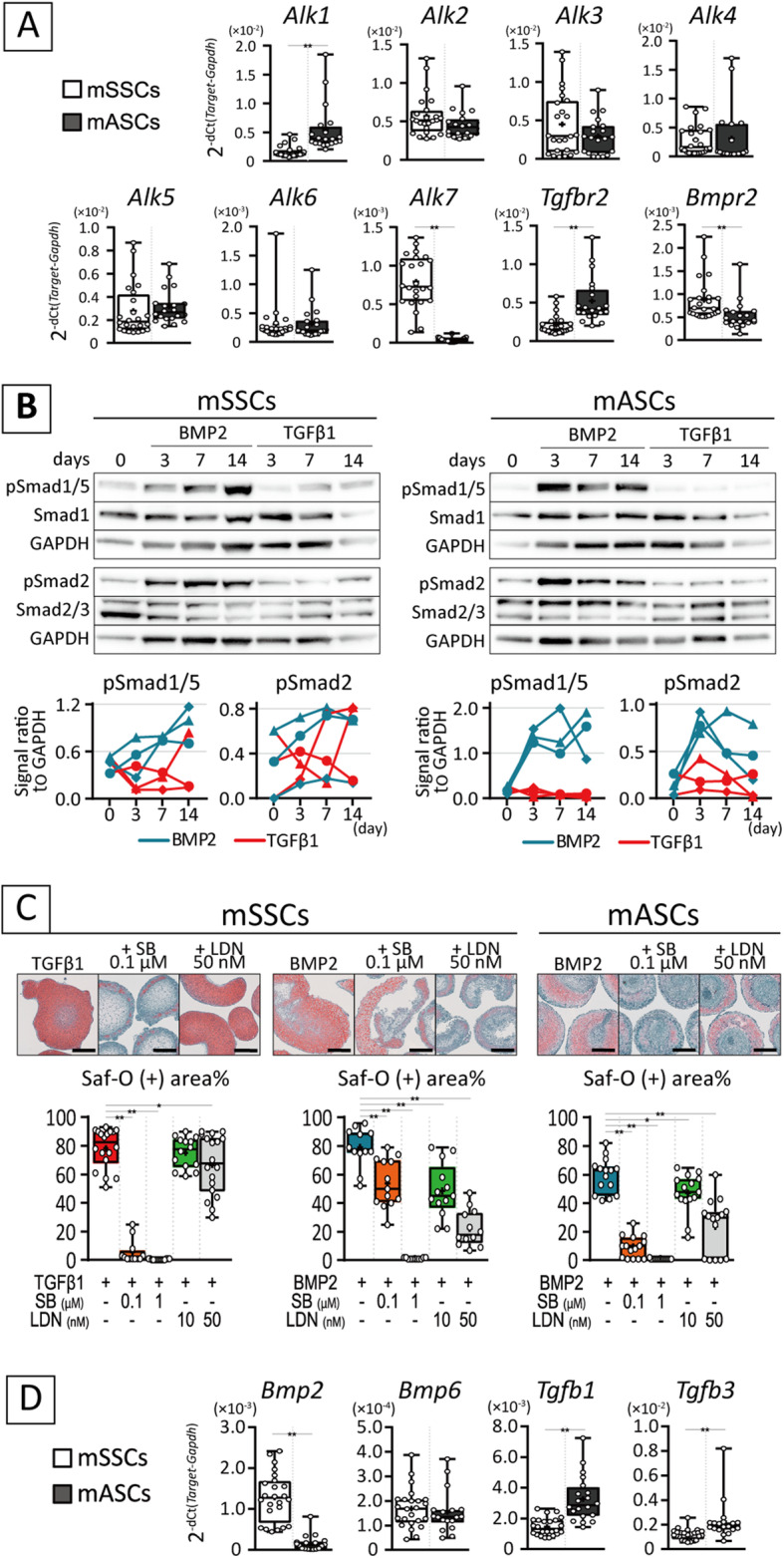
TGFβ superfamily receptors and ligands in mASCs and mSSCs during chondrogenic culture. **A, D** Gene expression levels for TGFβ superfamily receptors and ligands in day 0 pellet of mSSCs and mASCs. Data are collected from over three independent lots and presented as a box plot with dot plot. Significance was assessed using Student’s unpaired *t* test (**, *p* < 0.01). **B** Western blot analysis of Smads in mSSCs and mASCs in chondrogenic culture. Representative images from two or three lots are shown. Signal intensity of pSmad1/5 and pSmad2 relative to GAPDH are shown as line graph which shape of points represent individual cell lots. **C** Saf-O images with the box plot for their positive stained area of histology from mSSCs and mASCs cultured with BMP2, TGFβ1, SB-431542 (SB), and/or LDN-193189 (LDN) for 14 days. Scale bars = 500 μm. Significance was assessed using one-way ANOVA with Dunnet test (*, *p* < 0.05; **, *p* < 0.01)

To elucidate the distinct outcome in response to TGFβ1 among cell types, the expression of their specific receptors was confirmed. Among TGF receptor superfamilies, Alk1 and Alk7 were remarkably higher in mSSCs and mASCs, respectively. However, Alk5 which is a major TGFβ type 1 receptor, and Tgfbr2 were comparable in mSSCs and mASCs. There was no difference in Alk2, Alk3, Alk4, Alk6, and Bmpr2 (Fig. 3A). Furthermore, the levels of most receptors did not dynamically alter in 2 weeks of chondrogenic culture (see Additional file 9A). Western blotting analysis showed that during short-term stimulation, Smad1/5 and Smad2 were activated in response to BMP2 and TGFβ1, respectively, in both cell types according to conventional theory (see Additional file 10). Thus, mASCs and mSSCs were comparable in terms of the expression of TGFβ receptors as well as of receptors mediating Smads phosphorylation.

Conversely, in chondrogenic pellet culture, TGFβ1 did not affect either Smad1/5 and Smad2 pathway in mASCs, while it temporally activated Smad2 and Smad1/5 in some batches of mSSCs with obscure trends (Fig. 3B, see Additional file 11). Notably, the activation of Smad2 was marked with the presence of BMP2 as well as Smad1/5 in both cell types. This was supported by the fact that SB-431542, an Alk5 inhibitor, strikingly abrogated chondrogenesis induced not only by TGFβ1 but also by BMP2 (Fig. 3C). Although TGFβ signal is associated with cell growth and survival, its inhibition led adipogenesis not cell death (see Additional file 12).

LDN-193189, a BMP receptor (Alk2 and Alk3) inhibitor, also weakened TGFβ1-induced chondrogenesis of mSSCs to a lesser extent than it weakened BMP2-induced chondrogenesis (Fig. 3C) as 10-fold higher dose of LDN-193189 was required to inhibit TGFβ1-induced chondrogenesis (see Additional file 13). Considering the activation of Smad2 during BMP2 stimulation, it is possible that autocrine ligand expression contributed to chondrogenesis. Regarding endogenous chondrogenic growth factors, Bmp2 was expressed higher in mSSCs than mASCs whereas TGFβ1/3 were expressed at comparable levels (Fig. 3D). Bmp4 and Bmp7 were barely expressed in two cell types (data not shown). Tgfb1/3 were constant or marginally upregulated in chondrogenesis, and Bmp2 and Bmp6 were upregulated in BMP2 stimulation toward the end of induction, similar to Col10a1 (see Additional file 9B).

Based on the above in vitro findings, it can be inferred that TGFβ receptor-mediated signal was indispensable for chondrogenesis. However, the output in response to TGFβ could not be determined in their profiles for receptors and ligands.

### Distinct chondrogenic conditions and outcomes between hASCs and hSSCs with variations among donors

To expand the scope of application of murine experiments in human somatic stem cells, the responses of hASCs and hSSCs to chondrogenic growth factors were evaluated in vitro.

**Fig. 4:**
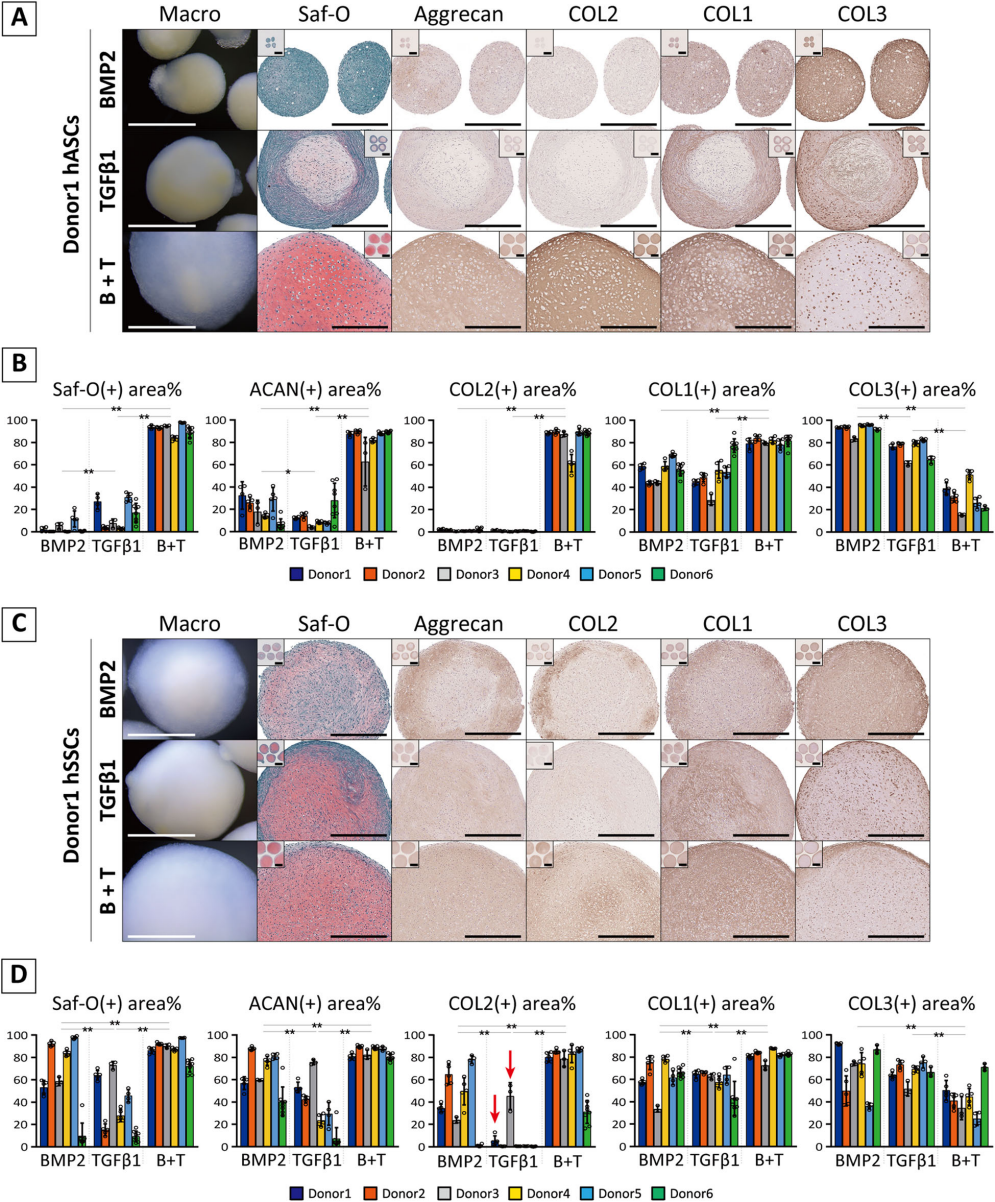
Chondrogenic potential of hASCs and hSSCs in the supplementation with BMP2 and/or TGFβ1. **A, C** Macroscopic view and histological images for chondrogenic pellet of cultured with BMP2, TGFβ1, or BMP2+ TGFβ1 (B + T) for 28 days. Representative data from six donors are shown. Scale bars = 1 mm (low magnified histology shown as inbox) and 500 μm (macro view and histological images). **B, D** Quantification of histological images. Data are expressed as a bar plot with a dot plot. Significance among chondrogenic factors was assessed using two-way ANOVA with post hoc Turkey HSD test (*p < 0.05, **p < 0.01)

When hASCs were cultured with BMP2 or TGFβ1 alone, weak staining for Saf-O and aggrecan was recognized in the formed tissues in some cases but COL2 was completely absent (Fig. 4A). Instead, COL1/3 were abundant in their tissues. Their chondrogenic potential could be demonstrated with use of the combination of BMP2 and TGFβ1, as evident from the development of proteoglycan- and type 2 collagen-enriched translucent tissue. With increased cartilaginous matrices, COL3 was restricted to the peripheral zone; however, COL1 was observed, similar to that in other single treatments. These results were consistently observed in all six donors (Fig. 4B).

In hSSCs, BMP2 + TGFβ1 stably induced chondrogenesis in all donors similar to hASCs, whereas a distinct pattern for chondrogenic response to single stimulation was uniquely exhibited among the donors (Fig. 4C, D). When hSSCs were cultured with BMP2 alone, different grades of chondrogenesis were observed among donors: partially intense staining for chondrogenic markers was observed in 3/6 donors (D1, D3, and D4) and entire intense staining in 2/6 donors (D2 and D5) within developed tissues. Conversely, TGFβ1 alone induced proteoglycan synthesis, as observed in Saf-O and immunohistochemistry for aggrecan. Nevertheless, COL2 was recognized only in 2/6 donors (D1 and D3). Correlation analysis supported the inconsistency of the relationship between proteoglycans and COL2 (see Additional file 14A). Of note, all tissues developed from hASCs and hSSCs clearly contained COL1/3 under all given chondrogenic conditions. COL3 tended to be weakened in the specimen with intense staining for COL2; however, there was no negative correlation between COL1 and COL2 (see Additional file 14B and C).

**Fig. 5:**
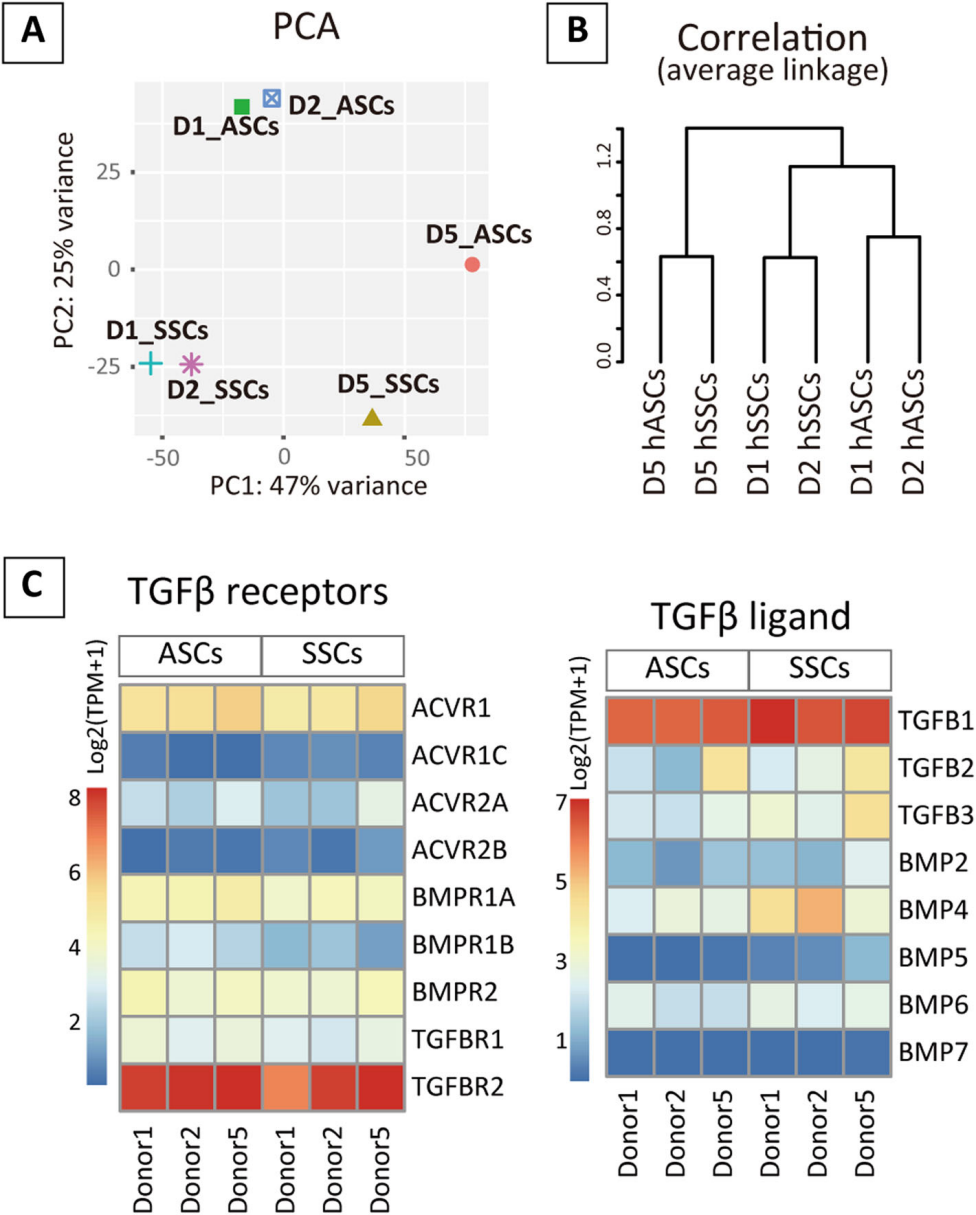
Gene profiling of hASCs and hSSCs before chondrogenic culture. **A, B** Principal component analysis (PCA) and cluster dendrogram based on gene profile. **C** Heatmap plotted with log2(TPM + 1) value of TGFβ superfamily receptors and ligands

Interestingly, the chondrogenic potential based on the response to BMP2 was not representative of the chondrogenic response to TGFβ1 (see Additional file 15). Thus, unique chondrogenic responses were observed between cell types and donors, although principal component analysis plot and hierarchical correlation analysis of mRNA-seq data showed low consistency in gene profiles between hASCs and hSSCs (Fig. 5A, B, see Additional file 16 and 17). The expression of receptors and ligands of the TGFβ superfamily was comparable among cell types and donors (Fig. 5C). Moreover, no specific enrichment was observed in multiple gene sets related to TGFβ signaling, proteoglycan, and chondrogenesis/cartilage development (see Additional file 18).

Thus, hASCs and hSSCs required distinct chondrogenic conditions beyond those required by mouse somatic stem cells. Moreover, the fibrous components were more abundant in developed tissues in human stem cell cultures, which characteristically resembled fibrocartilage. However, similar to that in mouse stem cells, the chondrogenic potential could not be predicted based on receptor/ligand expression.

### The cell response to TGFβ1 but not BMP2 reflected in vivo reaction of human somatic stem cells

To investigate the in vivo chondrogenic potential of human stem cells, hASCs and hSSCs were transplanted into the osteochondral defect model using SCID mice without pre-chondrogenic induction and evaluated at 4 weeks.

**Fig. 6:**
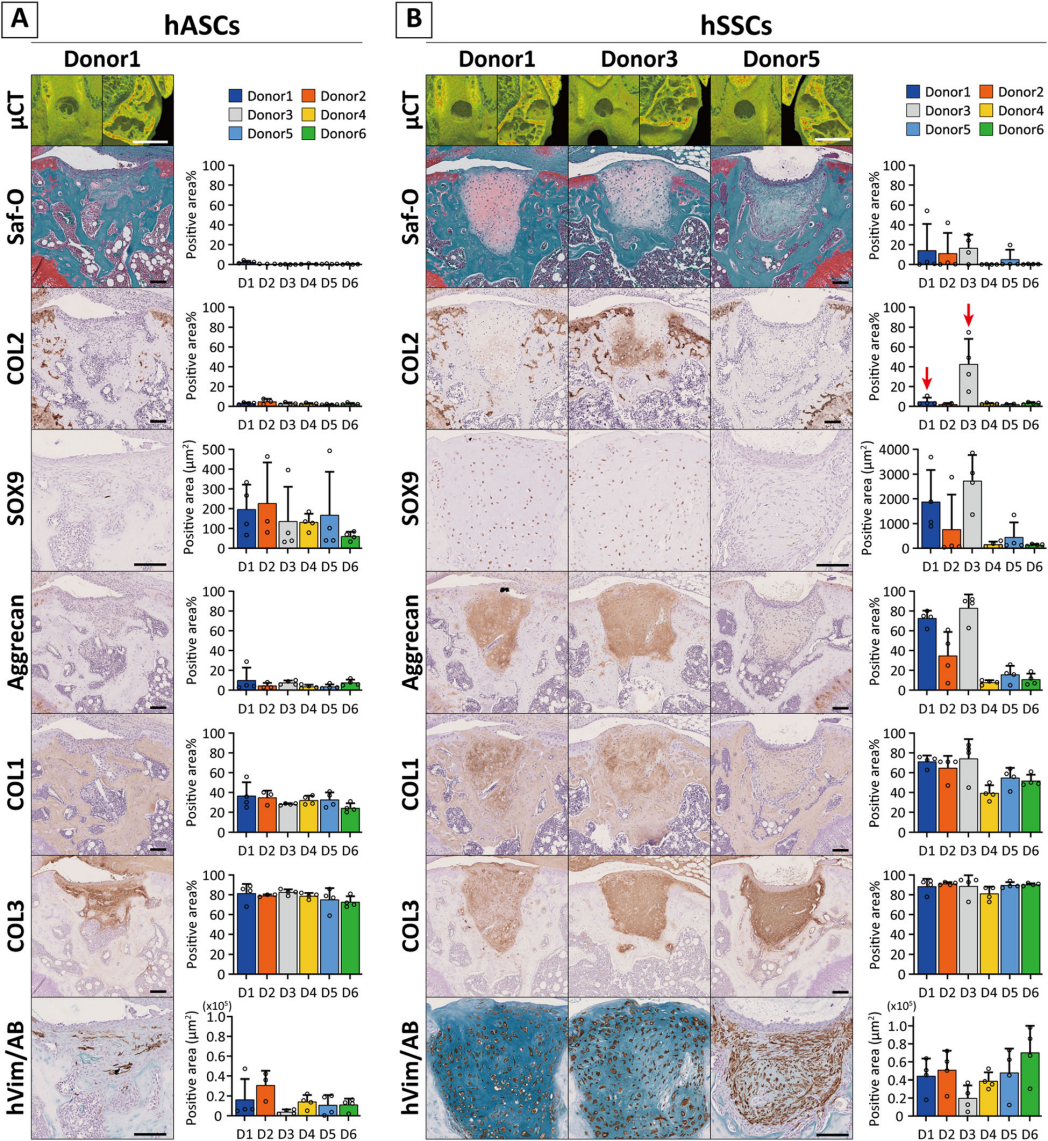
In vivo chondrogenic potential of hASCs and hSSCs in SCID mice osteochondral model. **A, B** Micro CT images and histological images with the qualification for their positive stained area in new formed tissues at day 28 after cell transplantation. Representative images are shown (*N* = 4 knees for each cells). Values are expressed as a bar plot with a dot plot. Scale bars = 1 mm (μCT) and 100 μm (histological images)

In case of hASC transplantation, the subchondral bone was partially regenerated, but the surface zone was filled with fibrous tissues composed of COL1/3, and no cartilaginous matrices were detected in all donors (Fig. 6A, see Additional file 19). hSSC transplantation revealed that defects did not contain bony tissues; instead, newly formed tissue filled out the defect space in all specimens. Among them, the formation of cartilaginous tissue containing proteoglycan, partial COL2, and SOX9 expressing cells was occasionally recognized in D1 (*N* = 1/4) and D3 (*N* = 4/4), which exhibited a chondrogenic response to TGFβ1 in vitro (Fig. 6B, see Additional file 20). Notably, as indicated in vitro, the fibrous component, COL1/3, was present in the entire defect site. Immunohistochemistry for human vimentin showed that hASCs, without any specific differentiation, diffusely remained in tissues with granulation rich in hypercellularity and small vessels (Fig. 6A, see Additional file 21A). On the contrary, hSSCs were better engrafted within the osteochondral region even in cells with the lowest in vitro chondrogenic potential (Fig. 6B, see Additional file 21B). In some cases, the defect area in subchondral bone widened and/or spilled out into host area. Moreover, the erosion sometimes reached to the articular cartilage (see Additional file 21, arrow heads). TRAP activity indicated erosion at the border between the neo-tissue and surrounding bone and cartilage, whereas there was lesser erosion in the periphery of the neo-tissue enriched in aggrecan-positive matrices (see Additional file 21).

Collectively, the cell response to TGFβ1 but not BMP2 possibly presents the in vivo chondrogenic reaction in human somatic stem cells like mouse study. However, the engraftment rate was intrinsically different among cell types regardless of the chondrogenic potential (Fig. 6A, B). Furthermore, the local transplantation of cells with lower potential for in vivo chondrogenesis may lead to other side effects such as erosion of the bone and cartilage.

## Discussion

Our findings showed that the chondrogenic potential of somatic stem cells in vitro, which has been defined in BMP2-supplemented conditions, did not correlate with the reparative capacity for local transplantation therapy of the osteochondral defect in vivo. Moreover, this study is the first to demonstrate that the response to TGFβ1 in vitro represents the outcome after local transplantation, which may be a potential indicator to predict in vivo chondrogenesis. Although chondrogenic response of hSSCs had variety among donors, the test for chondrogenic response to TGFβ1 in vitro may provide a new indicator to select cell source from allogenic cell banks.

As the major limitation of rodents study, the subchondral bone defect must be employed for the purpose of studying only the cartilage region. However, our data showed lower reparability of the cartilage even in an osteochondral injury, whereas bone defect region was spontaneously regenerated with membranous ossification. For articular cartilage repair, cartilaginous callus formation is required to remodel the osteochondral region for endochondral development. As SSCs are known to induce host synovial tissues [35], location of the defect site is critical for mSSC transplantation because infiltration of the host synovial tissue was observed when the defect was close to the enthesis of crucial ligaments. Furthermore, transplanted mSSCs detected below the marrow area did not exhibit signs of chondrogenesis, and in case of synovial infiltration, vessel-like shapes were found (see Additional file 7). Thus, location and site-specific signals, including mechanical stress, may be necessary for in vivo chondrogenesis as chondrogenesis was not observed in the knee with patellar dislocation (data not shown) [36, 37].

Although we implied the importance of chondrogenic response to TGFβ1, the distinct chondrogenic result of each cell type could not be elucidated in the analysis of TGFβ superfamily receptors, endogenous ligands, and downstream pSmads. In fact, all cell types used here were considered to have a certain reaction to TGFβ with unique tissue formation, unlike that found in basal or BMP2-supplemented medium. Other studies have shown the inconsistencies in cellular receptor expression in in vitro chondrogenic activity [38, 39]. In general, TGFβs and BMPs bind their specific receptors; ALK5 is a type I receptor for TGFβs; ALK2, ALK3, and ALK6 are type I receptors for BMPs, which activate downstream Smads, Smad2/3, and Smad1/5/8, respectively. However, TGFβs are known to activate Smad1/5/8 pathway via binding to ALK1, a predominant BMP type I receptor [34], and BMPs can activate Smad2 pathway via binding to ALK5 [40]. The pathway that will be activated is determined by the abundance of receptors on the cell membrane and their affinity to ligands [41]. For example, TGFβ signaling in OA chondrocytes switch from the anabolic ALK5-Smad2/3 pathway to catabolic ALK1-Smad1/5/8 pathway due to disproportionate ALK1/ALK5 ratio, which accelerates cartilage destruction [42–45]. Using western blotting, we demonstrated that Smad activation in the order of hours and days were distinctly regulated. It is known that transcriptional targets of Smad2/3 dynamically change in the order of minutes and hours [46]. These results suggest that there are inadequate downstream gene markers to assess the activation of TGFβ signaling. Moreover, the non-Smad pathways also regulate cellular function and differentiation, including chondrogenesis [34, 47]. Thus, the entirety of TGFβ signaling from cue to output in each cell type is difficult to interpret; therefore, the chondrogenic response to TGFβs may not be predicted prior to chondrogenic culture.

BMP signaling is crucial for skeletogenesis, particularly for endochondral development. On the contrary, TGFβ signaling is crucial for joint development [48, 49] and homeostasis of articular cartilage [42, 50, 51]. In fact, TGFβ1 is constantly supplied to the synovial fluid from cartilage and synovium [52, 53]. Therefore, tests for response to TGFβ1 may reasonably predict cell fate after transplantation into joints. BMSCs undergo chondrogenesis with TGFβ treatment alone [17, 54] and are responsible for cartilage regeneration in focal cartilage defect using the bone marrow stimulation method [55]. We previously showed the in vivo chondrogenic potential of hBMSCs in a rat osteochondral model [22]. To further verify our proposal, we should have included hBMSCs in our analysis; however, as one of the limitations, it was difficult to prepare a sufficient number of hBMSCs from bone marrow effusion after osteotomy in TKA operation. Besides, we used subcutaneous fat from the knee area, which may have different characteristics from abdominal fat [56, 57]. Recently, medical waste-derived stem cells such as adipose tissue, synovium, umbilical cord, and dental pulp have been highlighted for stem cell therapy because of low additional invasion for tissue collection and their superior proliferative capacity compared with BMSCs [11, 58, 59]. Our data are limited and may not be applicable to other cell types; therefore, further study is required using other sources.

Unlike hASCs, there are various types of hSSCs among donors. This is because synovial tissue contains various components such as synovial lining, connective tissue, blood vessels, and adipose tissue, which are altered under pathologic conditions [60, 61]. Mochizuki et al. showed that adipogenic changes in human synovium caused low chondrogenic potential of their derivates [62], and Mizuno et al. demonstrated that each derivate of the synovial surface, stroma, and perivascular behaves as synovial fibroblasts in vitro with different chondrogenic potential under the combination of TGFβ3 and BMP2; among them, the perivascular-derived hSSCs exhibit the highest chondrogenic potential [63]. Furthermore, Sicasubramaniyan et al. showed that primary CD73^+^/CD90^−^ synovial cells showed a chondrogenic response to TGFβ1, but CD73^+^/CD90^+^ synovial cells required additional stimulation with BMP2 to undergo chondrogenesis [38]. The study also pointed a discrepancy in the expression of aggrecan and COL2 in chondrogenic culture of hSSCs, as shown herein as well as in our previous study [18]. Thus, conventional cell isolation from synovium without any selection yields low reproducibility, whereas our results may provide a new indicator for selecting a useful cell source.

It is known that synovial fibroblasts (SFs) contribute to cartilage and bone erosion in rheumatoid arthritis (RA) [64]. Given that cultured SFs have traditionally been studied in arthritis using the same isolation procedure used in therapeutic study, their erosive potential of hSSCs should be considered. Recent single-cell transcriptome analysis showed that inflammatory fibroblasts expressing TNFα, IL6, and IL1B as well as immune cells were found in OA synovium. Moreover, they also expressed several proteinases such as ADAMTSs and MMPs [65]. Tsuchiya et al. showed that the responses of OA-SFs to the stimulation of inflammatory cytokines were shared in RA-SFs, indicating the common potential for proinflammation in expanded cells derived from OA, RA [66], and probably healthy synovium. Furthermore, other groups demonstrated that hSSCs either from healthy subjects or OA patients could induce osteoclastogenesis from PBMCs in vitro [67]. These results support our data regarding the TRAP activity at the border of fibrous neo-tissues. Altogether, cultured synovial cells have two aspects as stem cells and disease-modifying fibroblasts. Regarding the purpose of regenerative therapy, further study will be needed to avoid the negative effects of hSSCs. These findings have not been reported in hASCs; however, the erosion of host tissue was also observed in cell transplantation. Our data indicate that local transplantation for chondral injuries using cells with low chondrogenic potential in vivo should be carefully considered.

There are several limitations in this study. Firstly, we did not verify the surface antigens and potential of cells for osteogenesis and adipogenesis, which are often used in MSC studies; however these have been confirmed in our previous studies [27, 68] or elsewhere with comparable culture methods [17, 59]. In fact, the criteria and terminology for MSCs have recently been reconsidered [69]. Therefore, we referred to somatic stem cells using the name of the source tissue in this study. With respect to repair in bone diseases, evaluation of the osteogenic capacity in vitro may be helpful; however, in vivo osteogenesis was not recognized in the cells studied herein. Secondly, we suggested that endogenous expression of TGFβ superfamilies may contribute during chondrogenesis, but it was only at the gene level. Further study will be needed to clarify at the protein levels, including dominant ligands for chondrogenesis. Thirdly, our human study was planned as donor matched comparison of two stem cells from OA patients; however, hSSCs had large variety among donors, resulting in an insufficient number to provide enough evidence using TGFβ responsible and non-responsible groups. To establish criteria, large number study focusing on human SSCs should be conducted including non-arthritis synovium. Finally, the endpoint of in vivo study was 3–6 weeks, which is insufficient to evaluate the actual fate of neo-tissue. Therefore, we could not declare that mSSC formed cartilaginous tissue can stably function as articular cartilage. Moreover, constituent changes in hSSCs forming fibrous or fibrocartilaginous tissues to form hyaline-like cartilage should be pursued further.

## Conclusion

Adequate chondrogenic factors driving chondrogenesis from somatic stem cells are intrinsically distinct among cell types and species. The response to TGFβ1 but not BMP2 may potentially represent the in vivo chondrogenic potential after transplantation into osteochondral defects. Our findings may help to establish an indicator to predict cell reparability for cartilage diseases prior to clinical use.

## Supplementary Information

**Additional file 1.** The information for donors.

**Additional file 2.** The information for immunohistochemistry.

**Additional file 3.** The information for qPCR primers.

**Additional file 4.** Chondrogenic pellet culture of mASCs and mSSCs in chondrogenic basal media at day 14. Representative data of macroscopic images and histology are shown.

**Additional file 5.** Gene expression of chondrogenic marker in the mASCs and mSSCs cultured with BMP2, TGFβ1, or their combination at day 14. Data are collected from over three independent lots and presented as a box plot with dot plot.

**Additional file 6.** Histological features for fibrous cartilage or ectopic cartilage development after transplantation with mouse ASCs.

**Additional file 7.** Extended data in mouse SSCs transplantation group without in vivo chondrogenesis. (A) In samples which defect site is close to enthesis, host synovial tissue infiltrated to defect site and inhibited in vivo chondrogenesis, but transplanted mSSCs existed within granulation tissue with unspecified state. (B) Synovial infusions must be observed in samples with patellar dislocation. Notably, IHC for GFP implies vessel like structure built by transplanted mSSCs. (C) In the samples with in vivo chondrogenesis, transplanted mSSCs existed with unspecified state in bone marrow region underneath neo cartilage.

**Additional file 8.** Saf-O and IHC for pSmad1 and pSmad2 of in vivo specimens of transplanted with mSSCs or mASCs. Representative data are shown. Scale bars = 100 μm.

**Additional file 9.** The alteration of gene expression of TGFβ superfamily receptors (A), ligands, and chondrogenic markers (B) during chondrogenic culture of mSSCs and mASCs. Data are expressed as Mean ± SD for each lot, and each shape of points represent individual lots.

**Additional file 10.** Western blot analysis of Smads in mSSCs and mASCs in two dimensions culture. Representative images from two lots are shown. Signal intensity of pSmad1/5 and pSmad2 relative to GAPDH are shown as line graph which shape of points represent individual cell lots.

**Additional file 11.** Extended images of western blotting during 14 days chondrogenic culture in other two lots of mASCs and mSSCs.

**Additional file 12.** Extended data and IHC for adipocyte marker, Perilipin in chondrogenic pellet cultured with BMP2, TGFβ1, and/or receptor inhibitors; SB-431542 and LDN-193189. Representative images are shown (A) and their measurement of positive stained are presented as a box plot (B).

**Additional file 13.** Inhibition of TGFβ1 induced chondrogenesis of mSSCs with higher dose of LND-193189. Representative Safranin-O staining from three independent lots is shown and their measurement of positive stained are presented as a box plot with dot plot.

**Additional file 14.** Pearson correlation analysis between measurements of histological images in chondrogenic pellet cultured with BMP2, TGFβ1, and their combination. (A) Correlation between proteoglycan and COL2 of hSSCs. (B,C) Correlation between fibrous marker and COL2 of hASCs. Each circle represents individual six donors, and circle size reflects standard deviation for the data plotted in Y axis.

**Additional file 15.** Histological images for donor3_hSSCs and donor5_hSSCs cultured with BMP2 or TGFβ1 for 28 days. Scale bars = 500 μm.

**Additional file 16.** RNA-seq analysis of hASCs and hSSCs in donor1, 2, and 5 before chondrogenic culture. Hierarchical clustering based on top 2000 genes with high standard deviation was shown.

**Additional file 17.** Heatmaps for genes collected from several gene set related in TGFβ receptor signaling, Proteoglycan, and Chondrogenesis/Cartilage development were shown.

**Additional file 18.** List of all genes with transcripts per million values of RNA-seq analysis.

**Additional file 19.** Micro-CT and histological images in the all samples transplanted with hASCs.

**Additional file 20.** Micro-CT and histological images in the samples transplanted with hSSCs. IHC images for COL2 with red frame have the area positively stained, and images with blue frame were except for quantification because of non-specific staining. IHC images for SOX9 with yellow frame represents the presence of SOX9 positive cells.

**Additional file 21.** (A, B) Representative images for IHC for hVimentin and TRAP staining from the samples transplanted with hASCs or hSSCs at day 28. Arrow heads indicate bone erosion spots. The number of TRAP positive spot at border between neo tissue and host bone or articular cartilage are expressed as a bar plot with a dot plot. Bars = 100 μm.

## Data Availability

Nucleotide sequence data reported are available in the DDBJ Sequenced Read Archive under the accession number DRA011462. Other data that support the findings of this study are available from the corresponding author upon reasonable request.

## Abbreviations

mASCs: Mouse adipose stem cells
mSSCs: Mouse synovial stem cells
hASCs: Human adipose stem cells
hSSCs: Human synovial stem cells
PBMCs: Peripheral blood mononuclear cells
BMP: Bone morphogenic protein
TGFβ: Transforming growth factor beta
Saf-O: Safranin-O
AB: Alcian blue
GFP: Green fluorescent protein
COL: Collagen
ACAN: Aggrecan
hVim: Human vimentin
TRAP: Tartrate-resistant acid phosphatase
SB: SB-431542
LDN: LDN-193189
OA: Osteoarthritis
RA: Rheumatoid arthritis
TKA: Total knee arthroplasty
