# Correction to: Divergence in chondrogenic potential between in vitro and in vivo of adipose- and synovial-stem cells from mouse and human

**DOI:** 10.1186/s13287-021-02556-7

**Published:** 2021-08-26

**Authors:** Ryota Chijimatsu, Satoshi Miwa, Gensuke Okamura, Junya Miyahara, Naohiro Tachibana, Hisatoshi Ishikura, Junya Higuchi, Yuji Maenohara, Shinsaku Tsuji, Shin Sameshima, Kentaro Takagi, Keiu Nakazato, Kohei Kawaguchi, Ryota Yamagami, Hiroshi Inui, Shuji Taketomi, Sakae Tanaka, Taku Saito

**Affiliations:** 1 Bone and Cartilage Regenerative Medicine, Graduate School of Medicine, The University of Tokyo, Tokyo, Japan; 2 Sensory and Motor System Medicine, Graduate School of Medicine, The University of Tokyo, Tokyo, Japan; 3 Orthopaedic Surgery, Osaka Rosai Hospital, Osaka, Japan; 4 Avenue CellClinic, Tokyo, Japan

## Correction to:** Stem Cell Research & Therapy** (2021) 12:405 https://doi.org/10.1186/s13287-021-02485-5

The original article [1] contained errors in the presentation of the authors’ names where by almost all authors’ names were mistakenly inverted.

This has since been corrected, and all authors’ names are now displayed appropriately.
