# Supplementary figures and images for: Divergence in chondrogenic potential between in vitro and in vivo of adipose- and synovial-stem cells from mouse and human
Source: Stem Cell Res Ther. 2021 Jul 15;12:405. doi: 10.1186/s13287-021-02485-5 (PMC8281654; doi:10.1186/s13287-021-02485-5)

## Slide 1
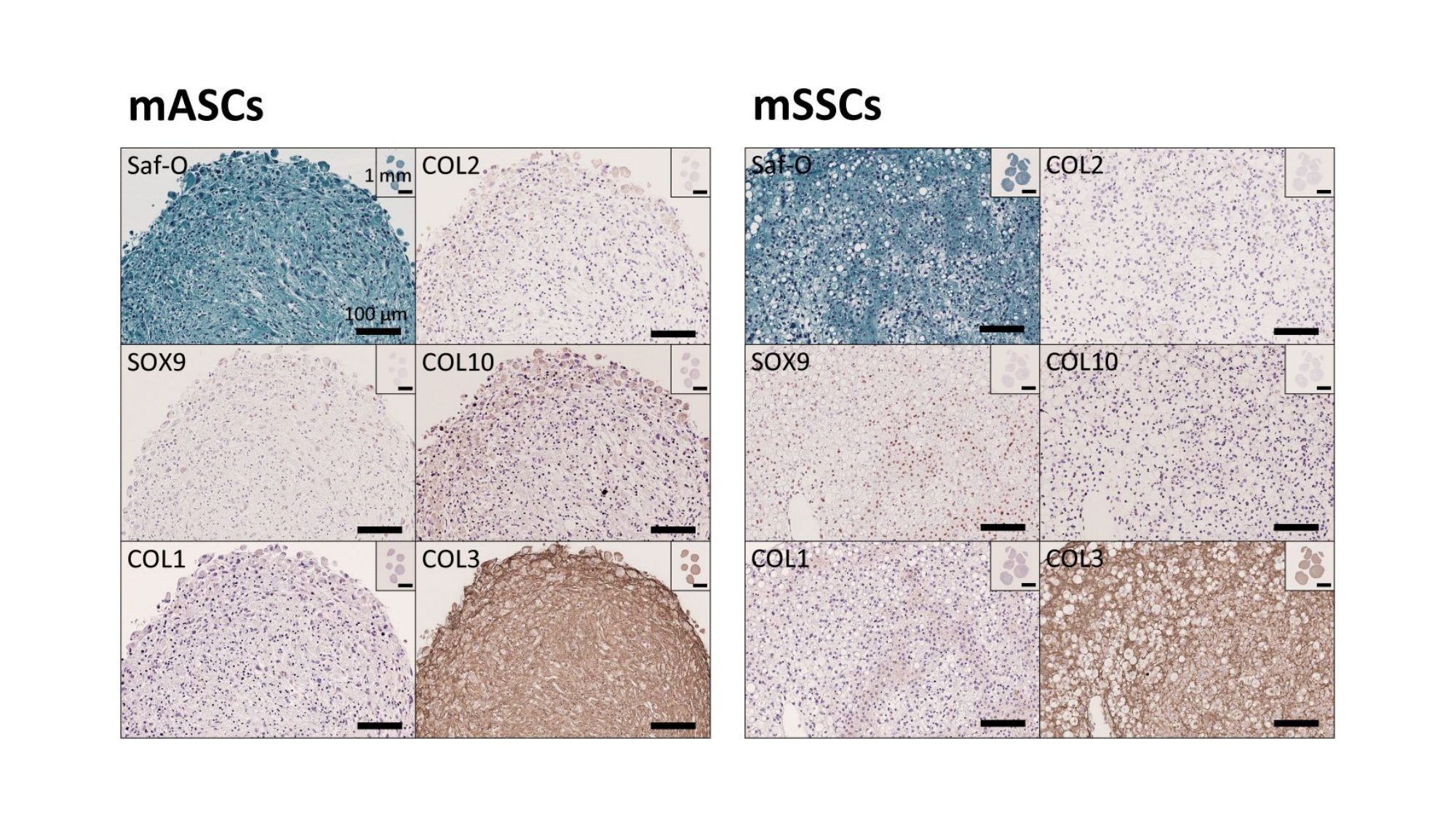

Supplement: Supplementary file 4 — Additional file 4. Chondrogenic pellet culture of mASCs and mSSCs in chondrogenic basal media at day 14. Representative data of macroscopic images and histology are shown. [file 13287_2021_2485_MOESM4_ESM.pptx]

## Slide 1
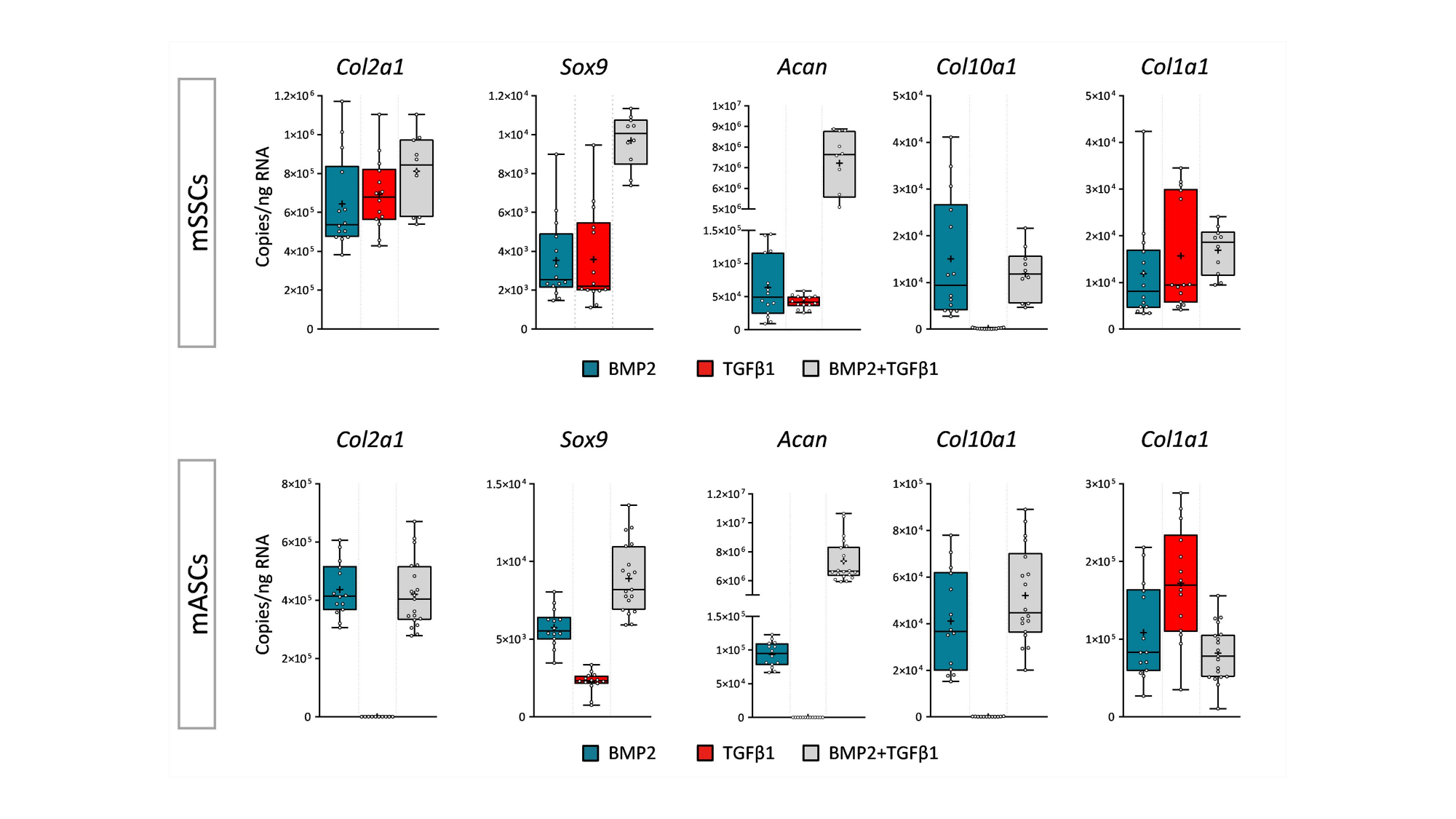

Supplement: Supplementary file 5 — Additional file 5. Gene expression of chondrogenic marker in the mASCs and mSSCs cultured with BMP2, TGFβ1, or their combination at day 14. Data are collected from over three independent lots and presented as a box plot with dot plot. [file 13287_2021_2485_MOESM5_ESM.pptx]

## Slide 1
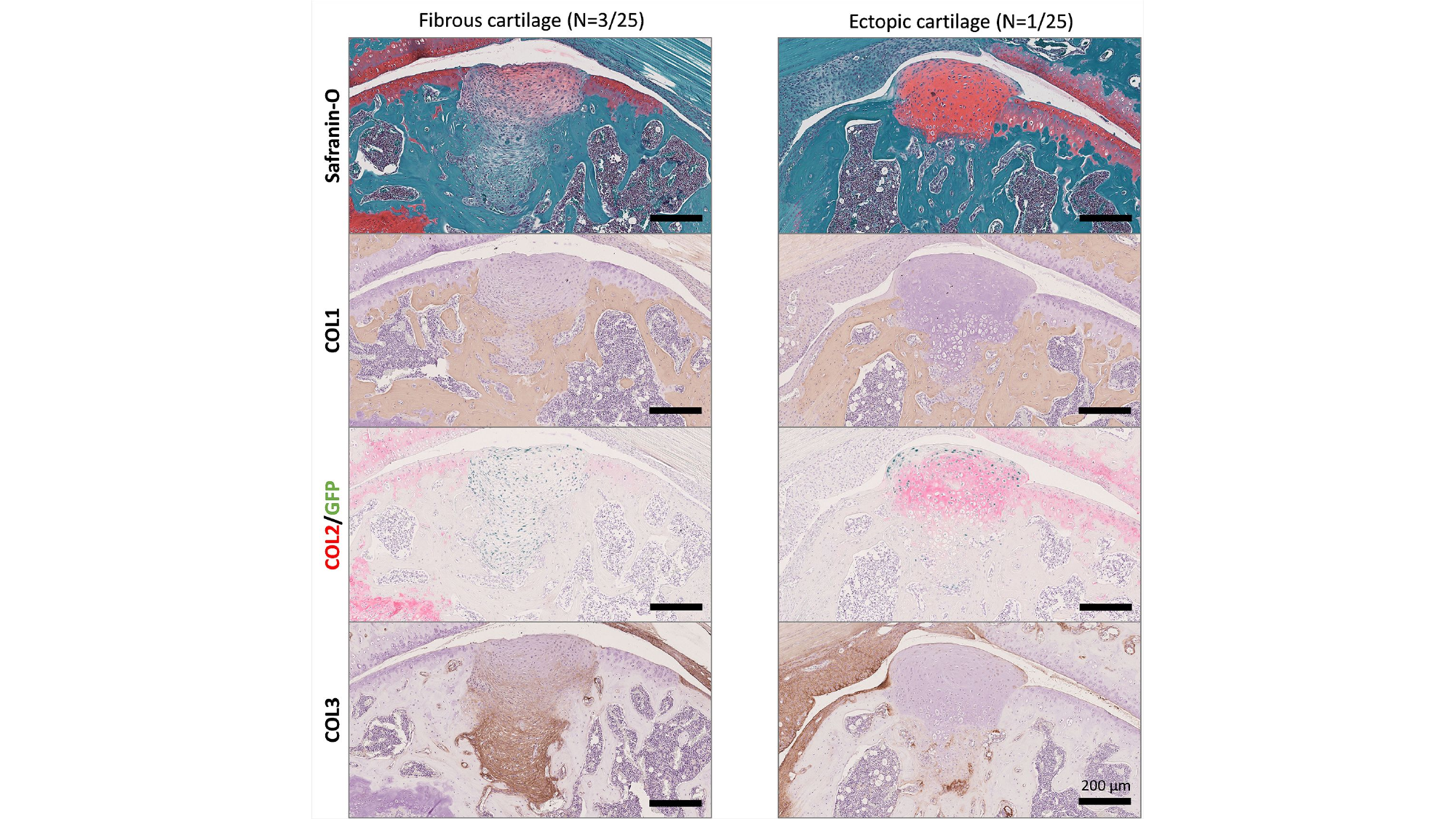

Supplement: Supplementary file 6 — Additional file 6. Histological features for fibrous cartilage or ectopic cartilage development after transplantation with mouse ASCs. [file 13287_2021_2485_MOESM6_ESM.pptx]

## Slide 1
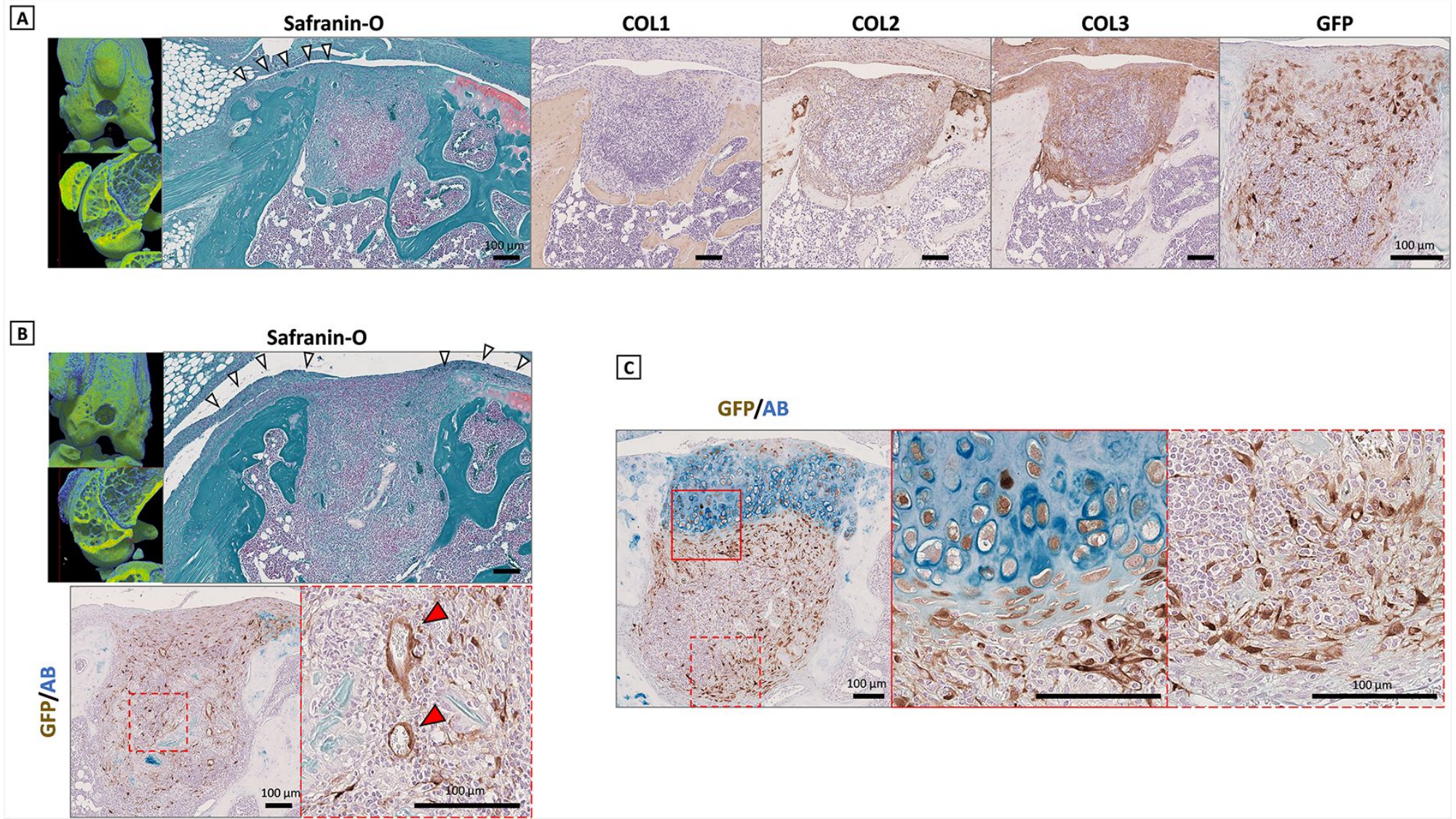

Supplement: Supplementary file 7 — Additional file 7. Extended data in mouse SSCs transplantation group without in vivo chondrogenesis. (A) In samples which defect site is close to enthesis, host synovial tissue infiltrated to defect site and inhibited in vivo chondrogenesis, but transplanted mSSCs existed within granulation tissue with unspecified state. (B) Synovial infusions must be observed in samples with patellar dislocation. Notably, IHC for GFP implies vessel like structure built by transplanted mSSCs. (C) In the samples with in vivo chondrogenesis, transplanted mSSCs existed with unspecified state in bone marrow region underneath neo cartilage. [file 13287_2021_2485_MOESM7_ESM.pptx]

## Slide 1
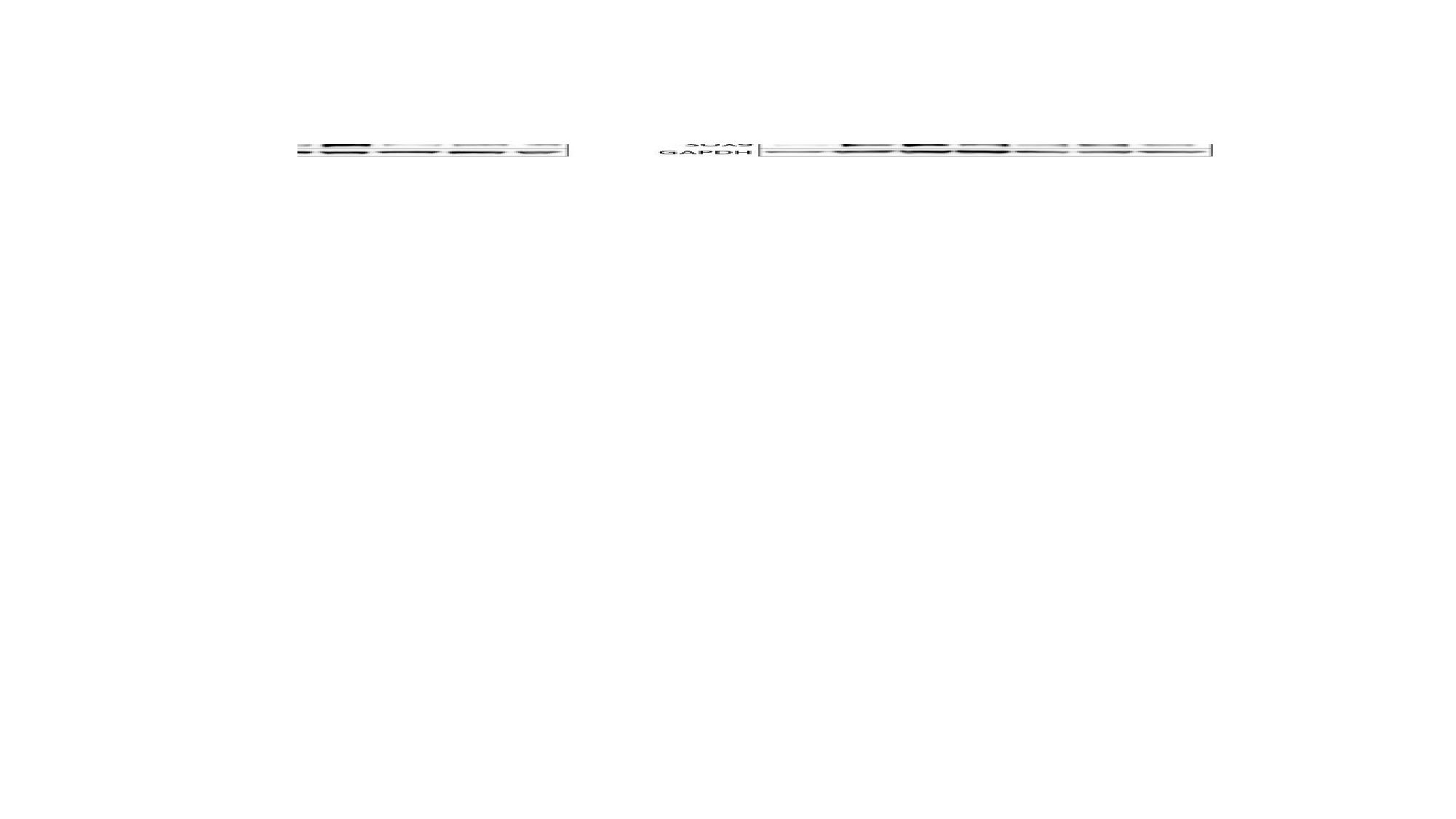

Supplement: Supplementary file 8 — Additional file 8. Saf-O and IHC for pSmad1 and pSmad2 of in vivo specimens of transplanted with mSSCs or mASCs. Representative data are shown. Scale bars = 100 μm. [file 13287_2021_2485_MOESM8_ESM.pptx]

## Slide 1
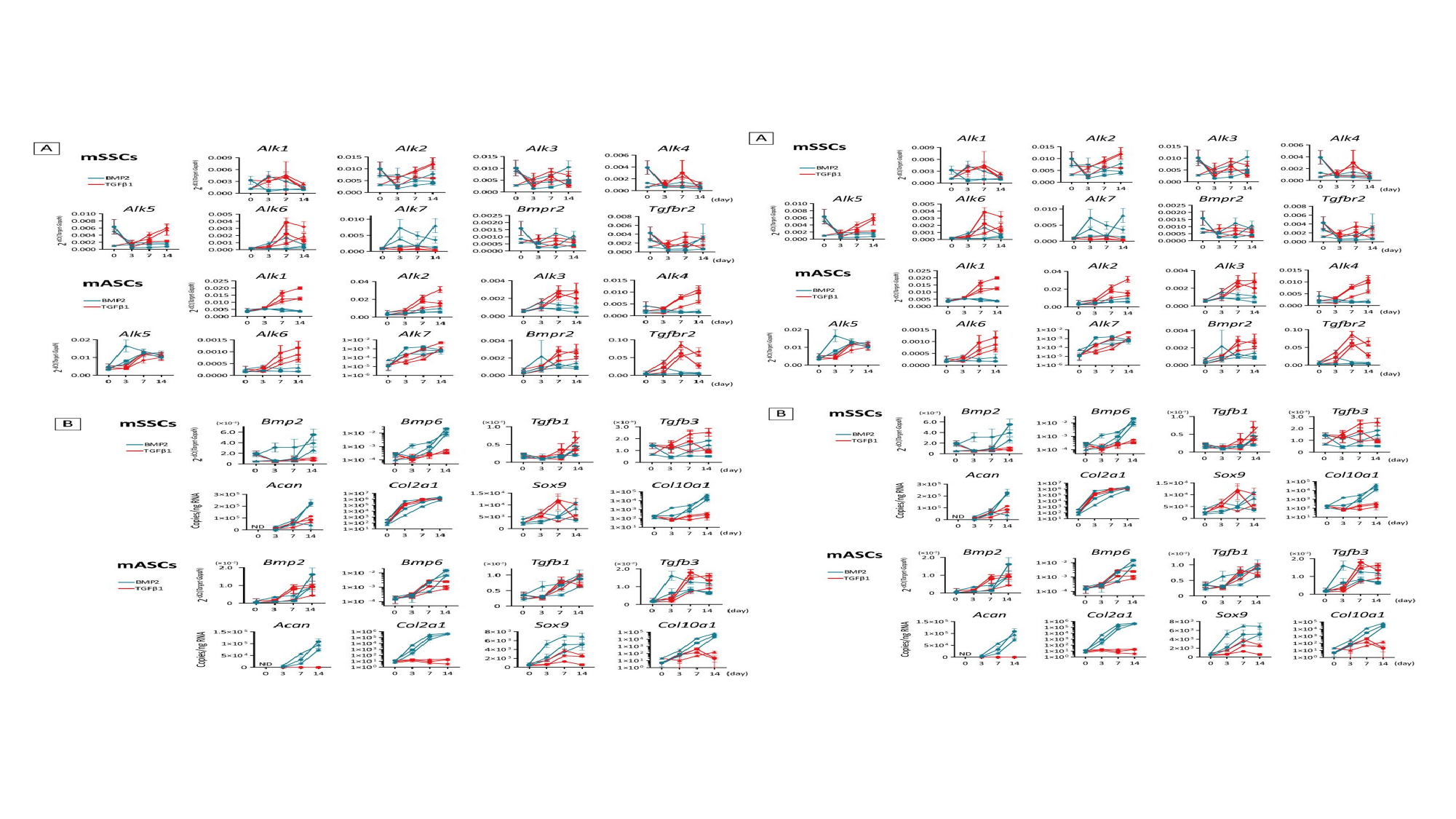

Supplement: Supplementary file 9 — Additional file 9. The alteration of gene expression of TGFβ superfamily receptors (A), ligands, and chondrogenic markers (B) during chondrogenic culture of mSSCs and mASCs. Data are expressed as Mean ± SD for each lot, and each shape of points represent individual lots. [file 13287_2021_2485_MOESM9_ESM.pptx]

## Slide 1
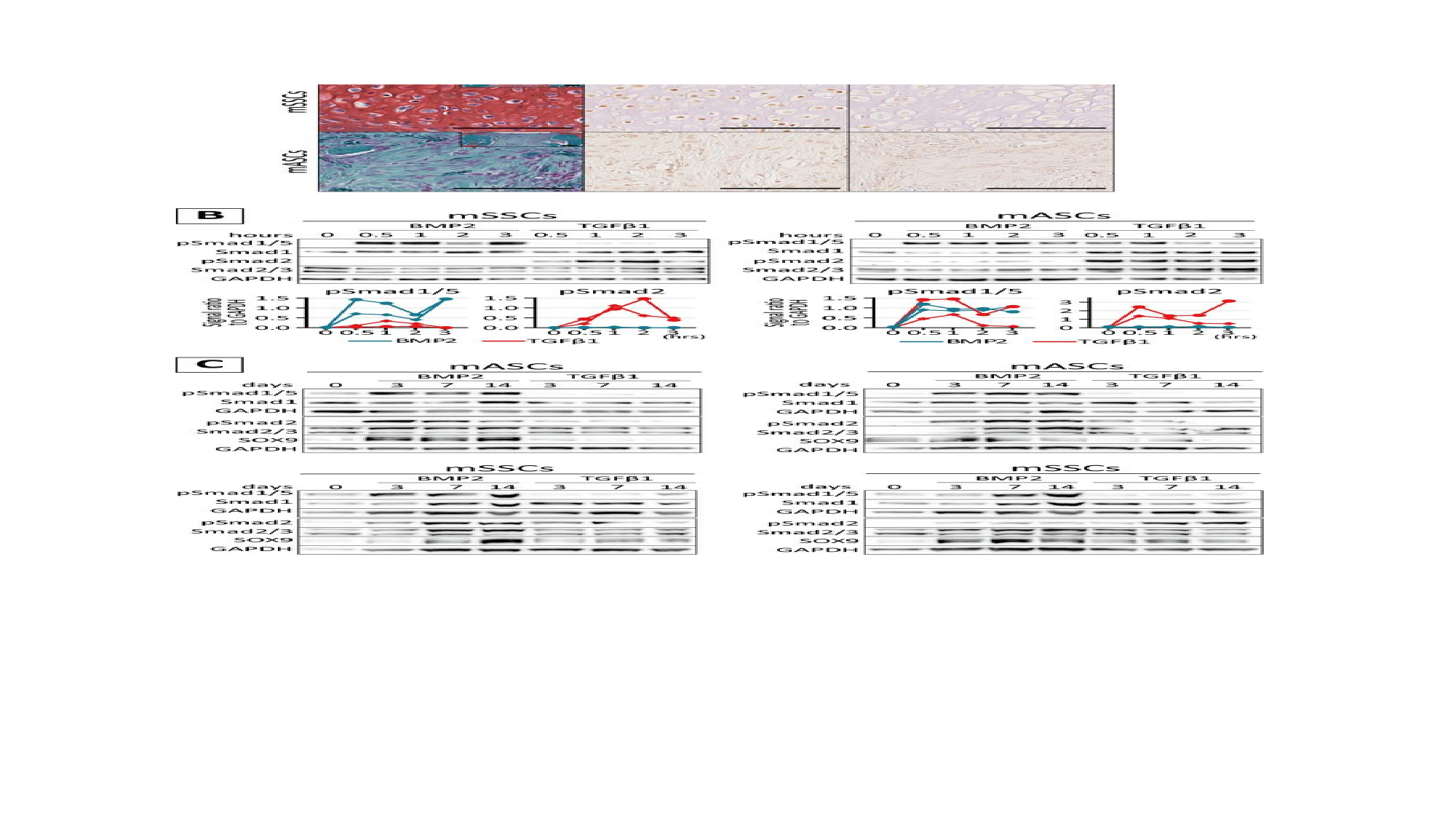

Supplement: Supplementary file 10 — Additional file 10. Western blot analysis of Smads in mSSCs and mASCs in two dimensions culture. Representative images from two lots are shown. Signal intensity of pSmad1/5 and pSmad2 relative to GAPDH are shown as line graph which shape of points represent individual cell lots. [file 13287_2021_2485_MOESM10_ESM.pptx]

## Slide 1
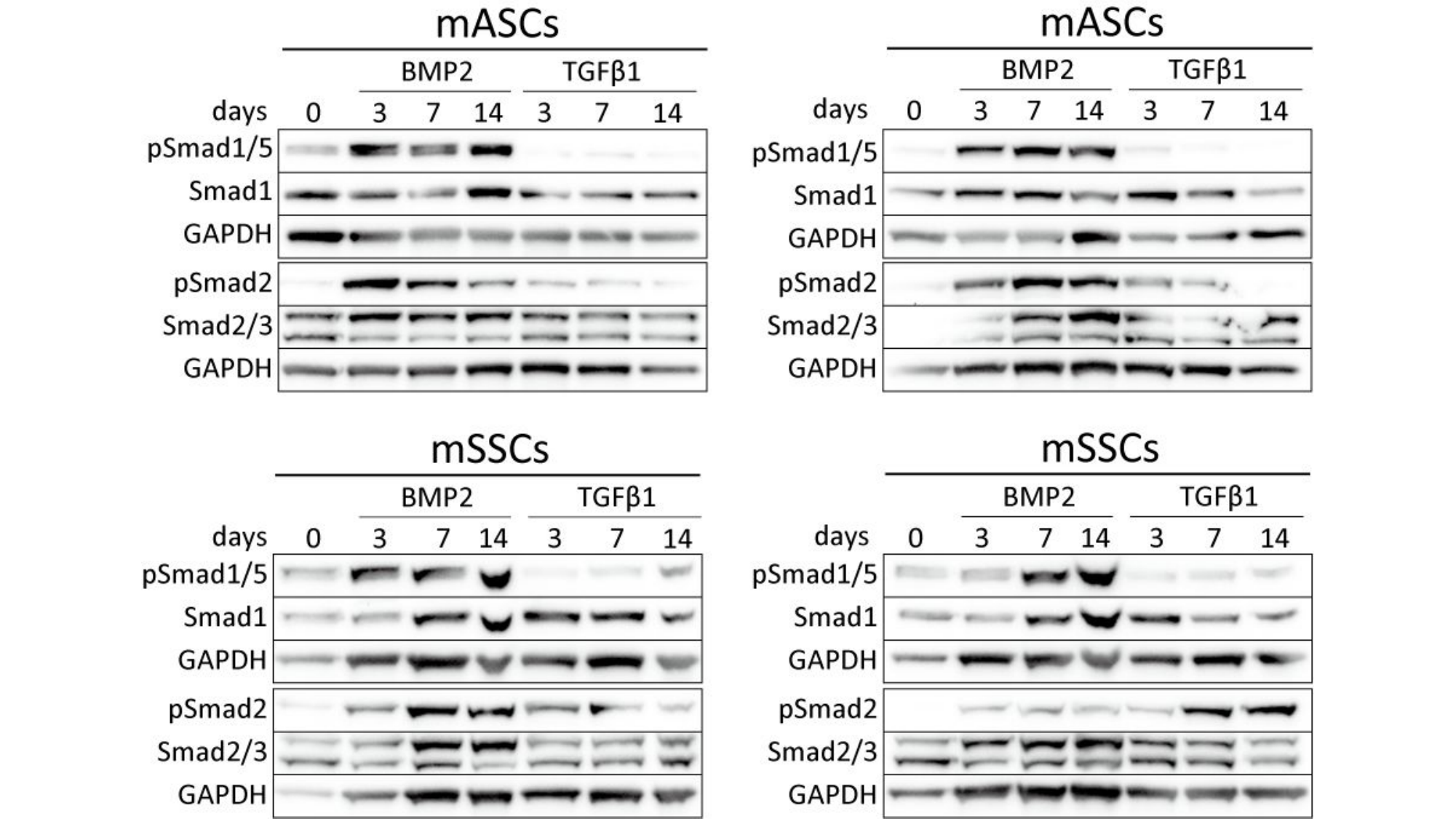

Supplement: Supplementary file 11 — Additional file 11. Extended images of western blotting during 14 days chondrogenic culture in other two lots of mASCs and mSSCs. [file 13287_2021_2485_MOESM11_ESM.pptx]

## Slide 1
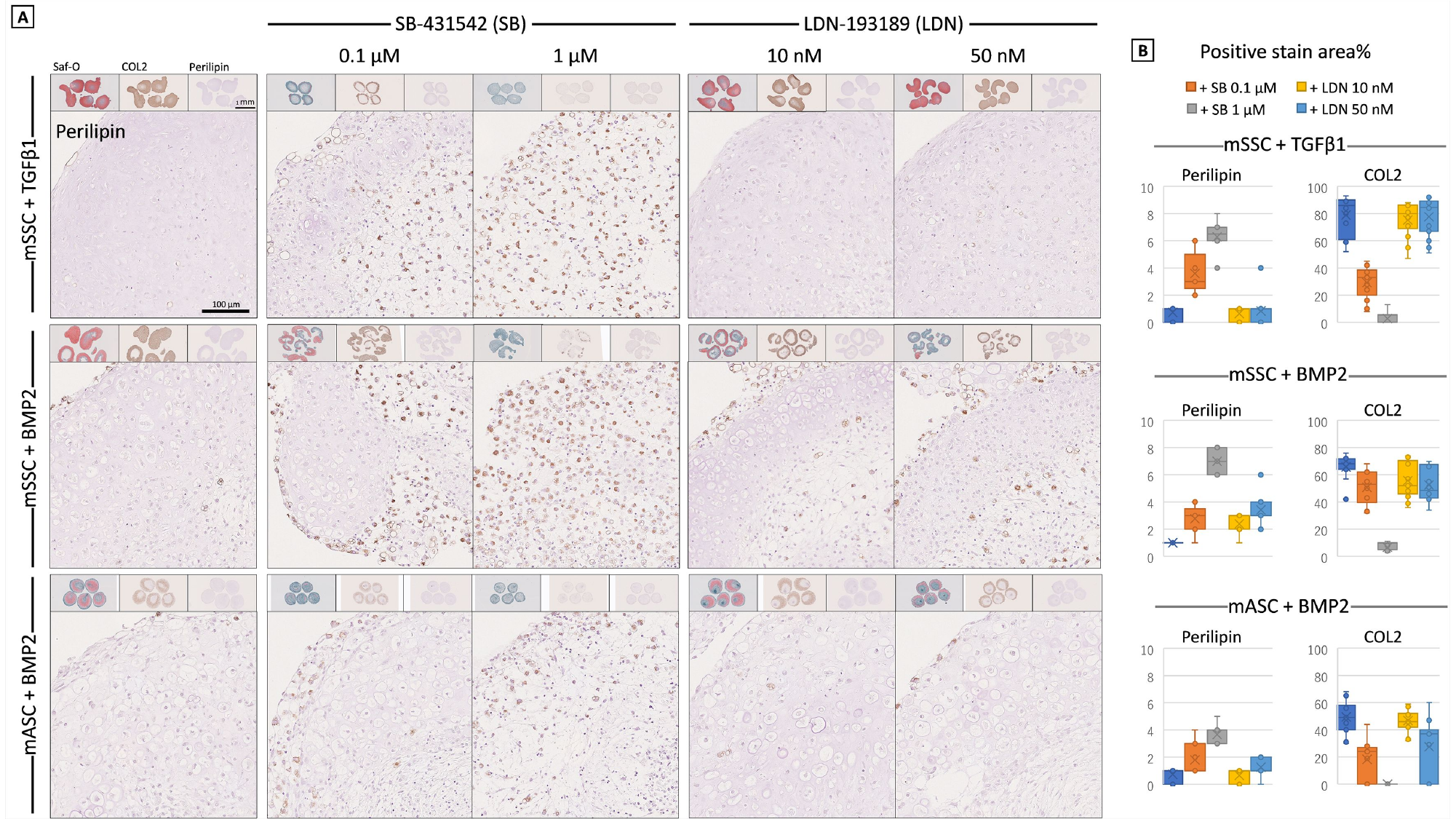

Supplement: Supplementary file 12 — Additional file 12. Extended data and IHC for adipocyte marker, Perilipin in chondrogenic pellet cultured with BMP2, TGFβ1, and/or receptor inhibitors; SB-431542 and LDN-193189. Representative images are shown (A) and their measurement of positive stained are presented as a box plot (B). [file 13287_2021_2485_MOESM12_ESM.pptx]

## Slide 1
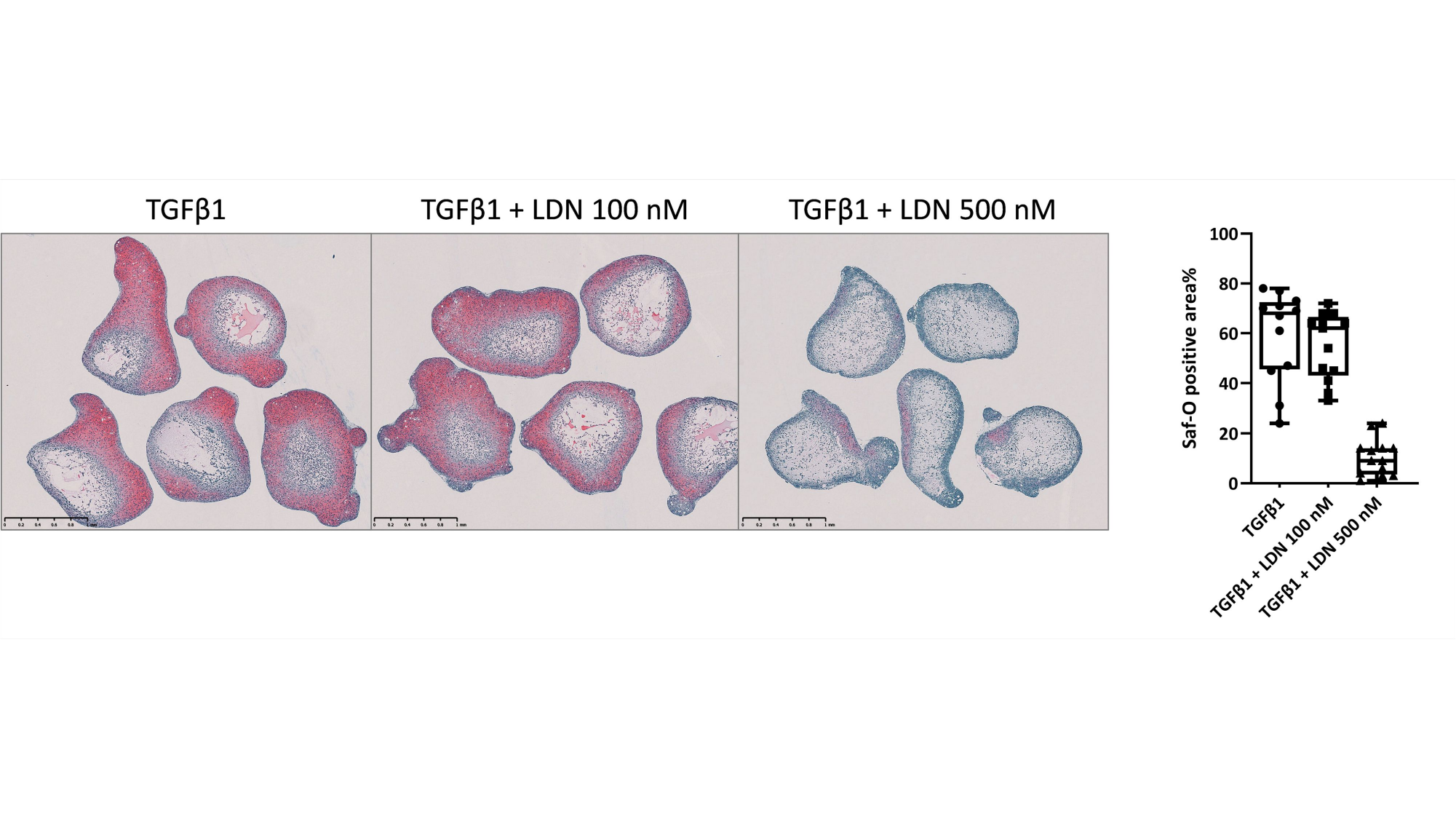

Supplement: Supplementary file 13 — Additional file 13. Inhibition of TGFβ1 induced chondrogenesis of mSSCs with higher dose of LND-193189. Representative Safranin-O staining from three independent lots is shown and their measurement of positive stained are presented as a box plot with dot plot. [file 13287_2021_2485_MOESM13_ESM.pptx]

## Slide 1
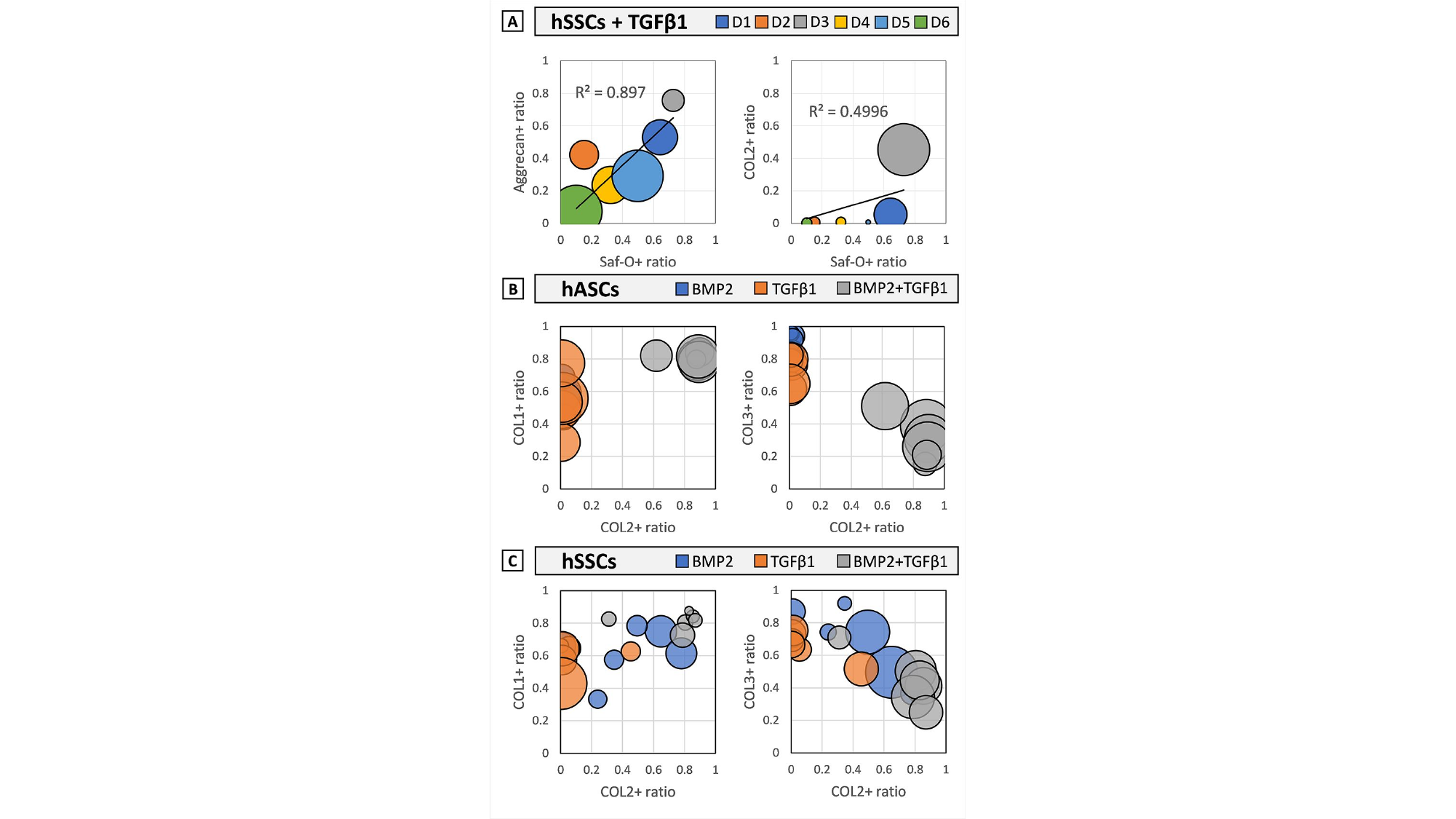

Supplement: Supplementary file 14 — Additional file 14. Pearson correlation analysis between measurements of histological images in chondrogenic pellet cultured with BMP2, TGFβ1, and their combination. (A) Correlation between proteoglycan and COL2 of hSSCs. (B,C) Correlation between fibrous marker and COL2 of hASCs. Each circle represents individual six donors, and circle size reflects standard deviation for the data plotted in Y axis. [file 13287_2021_2485_MOESM14_ESM.pptx]

## Slide 1
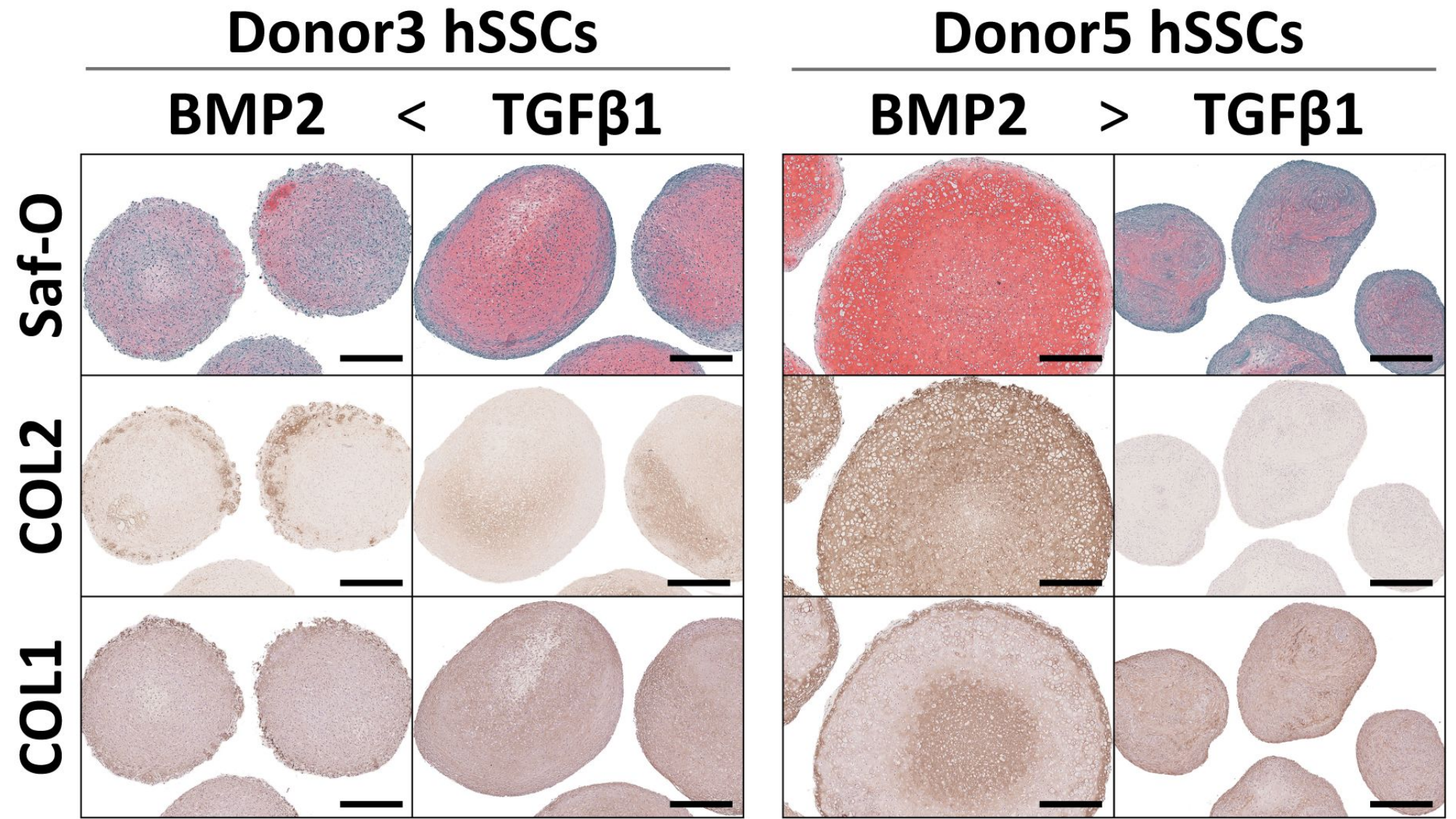

Supplement: Supplementary file 15 — Additional file 15. Histological images for donor3_hSSCs and donor5_hSSCs cultured with BMP2 or TGFβ1 for 28 days. Scale bars = 500 μm. [file 13287_2021_2485_MOESM15_ESM.pptx]

## Slide 1
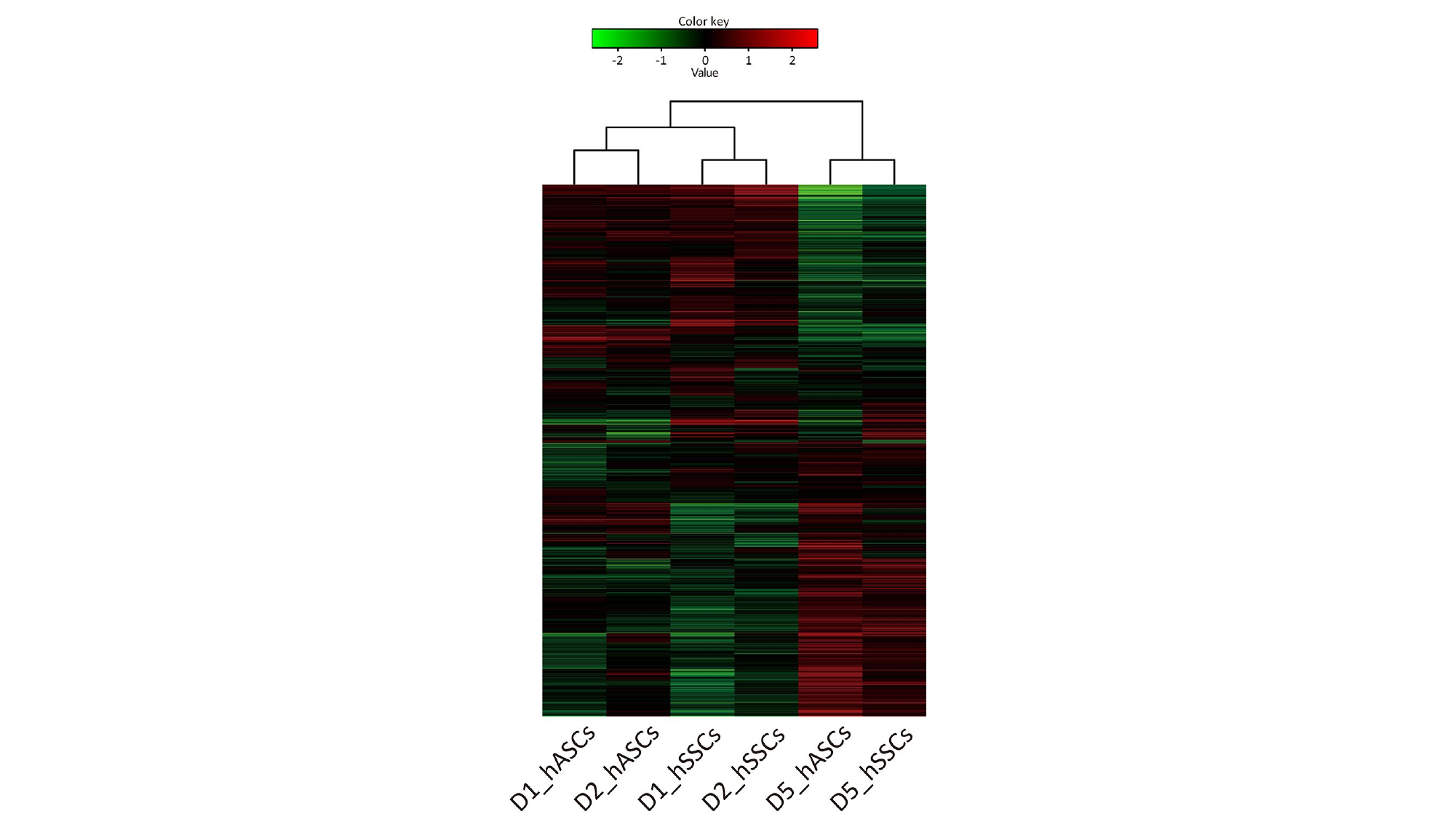

Supplement: Supplementary file 16 — Additional file 16. RNA-seq analysis of hASCs and hSSCs in donor1, 2, and 5 before chondrogenic culture. Hierarchical clustering based on top 2000 genes with high standard deviation was shown. [file 13287_2021_2485_MOESM16_ESM.pptx]

## Slide 1
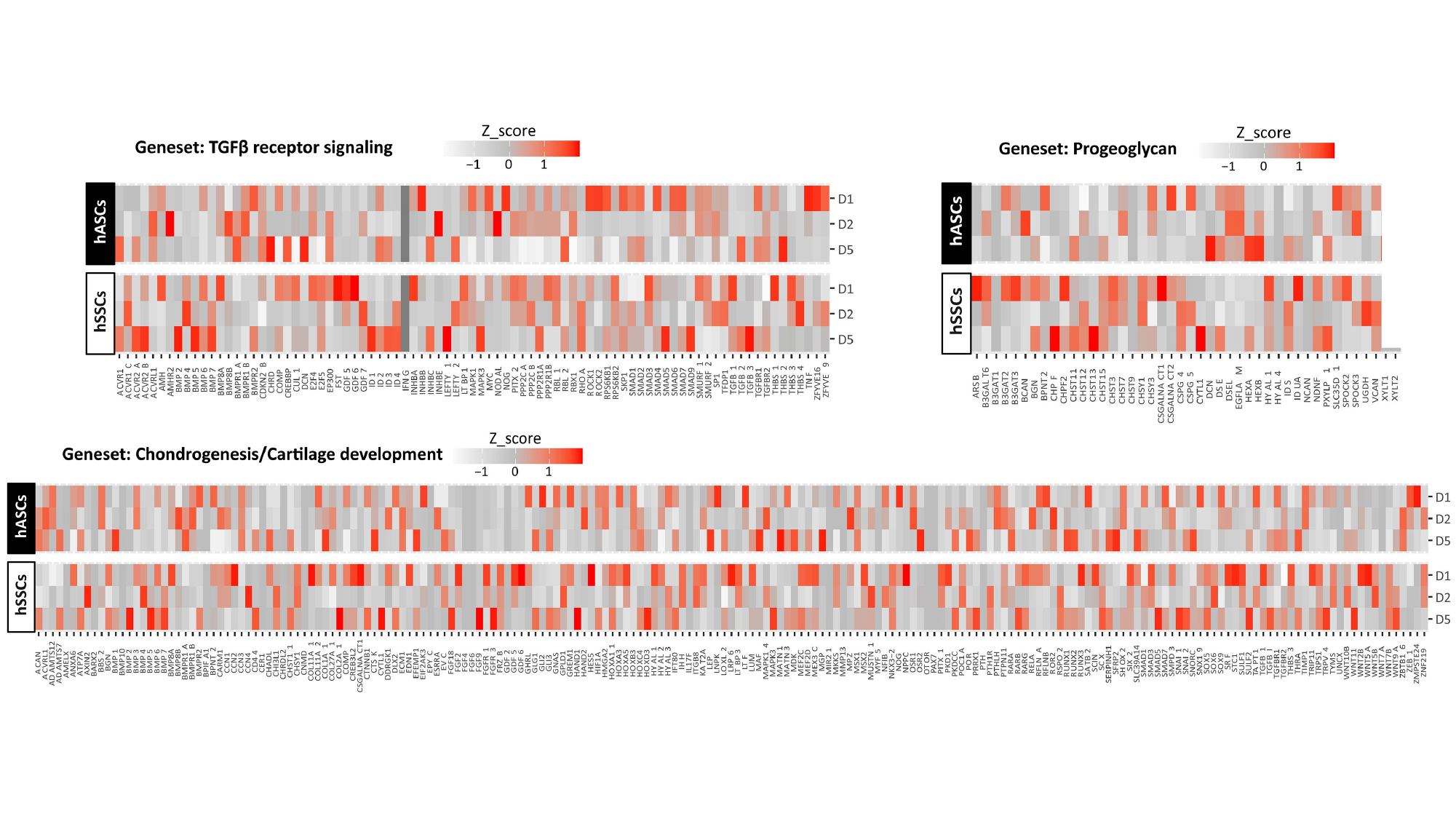

Supplement: Supplementary file 18 — Additional file 18. List of all genes with transcripts per million values of RNA-seq analysis. [file 13287_2021_2485_MOESM18_ESM.pptx]

## Slide 1
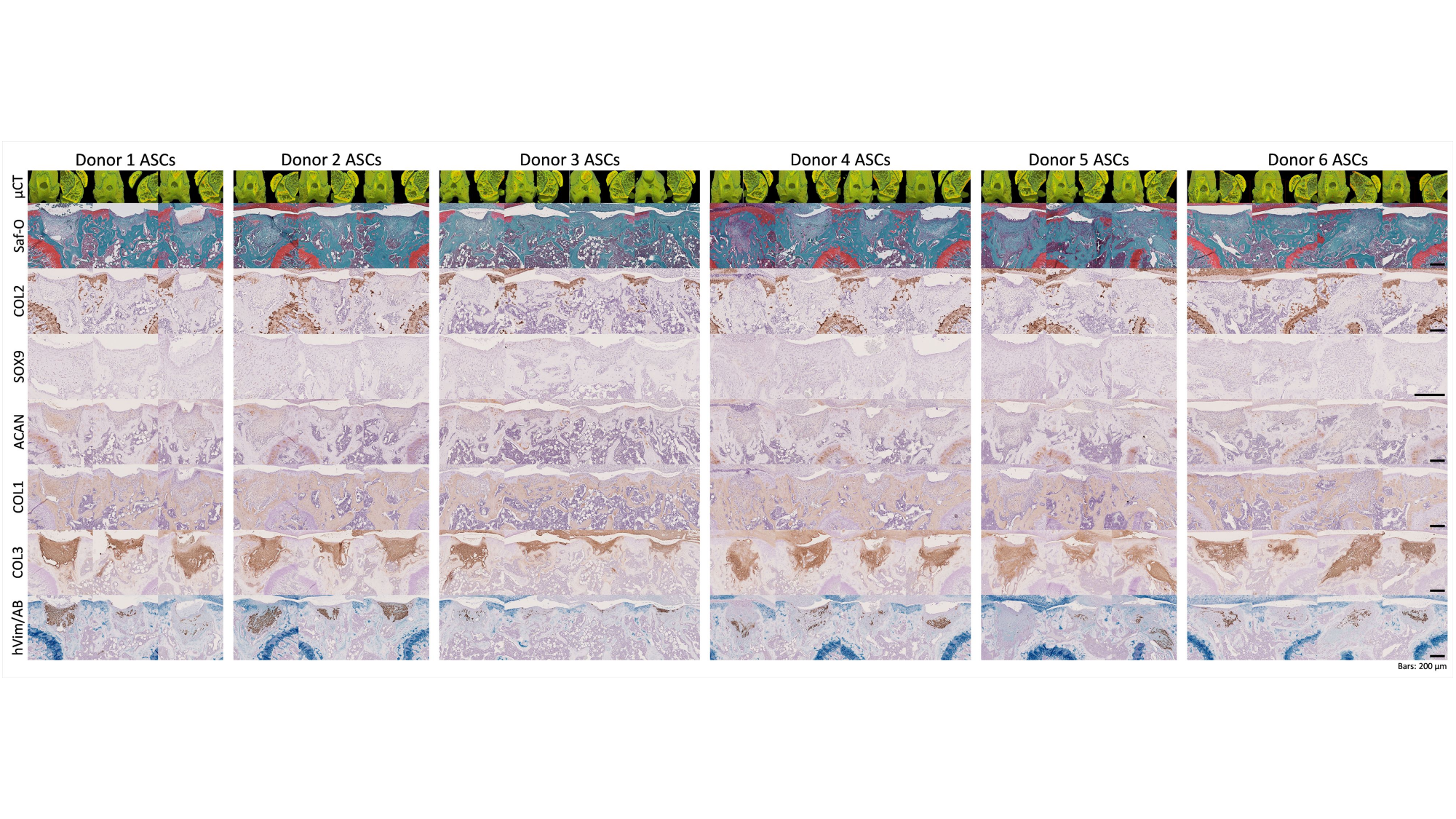

Supplement: Supplementary file 19 — Additional file 19. Micro-CT and histological images in the all samples transplanted with hASCs. [file 13287_2021_2485_MOESM19_ESM.pptx]

## Slide 1
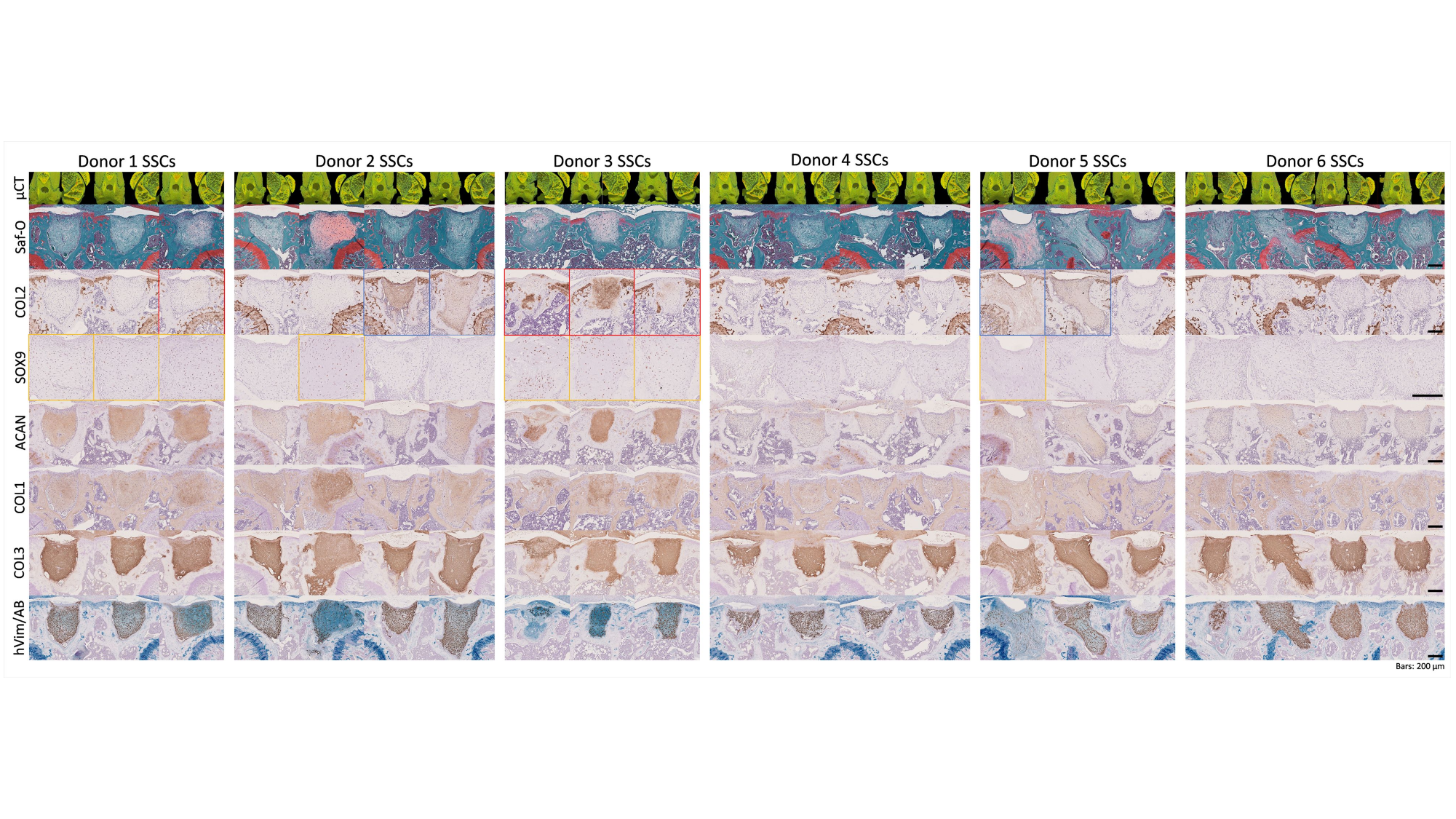

Supplement: Supplementary file 20 — Additional file 20. Micro-CT and histological images in the samples transplanted with hSSCs. IHC images for COL2 with red frame have the area positively stained, and images with blue frame were except for quantification because of non-specific staining. IHC images for SOX9 with yellow frame represents the presence of SOX9 positive cells. [file 13287_2021_2485_MOESM20_ESM.pptx]

## Slide 1
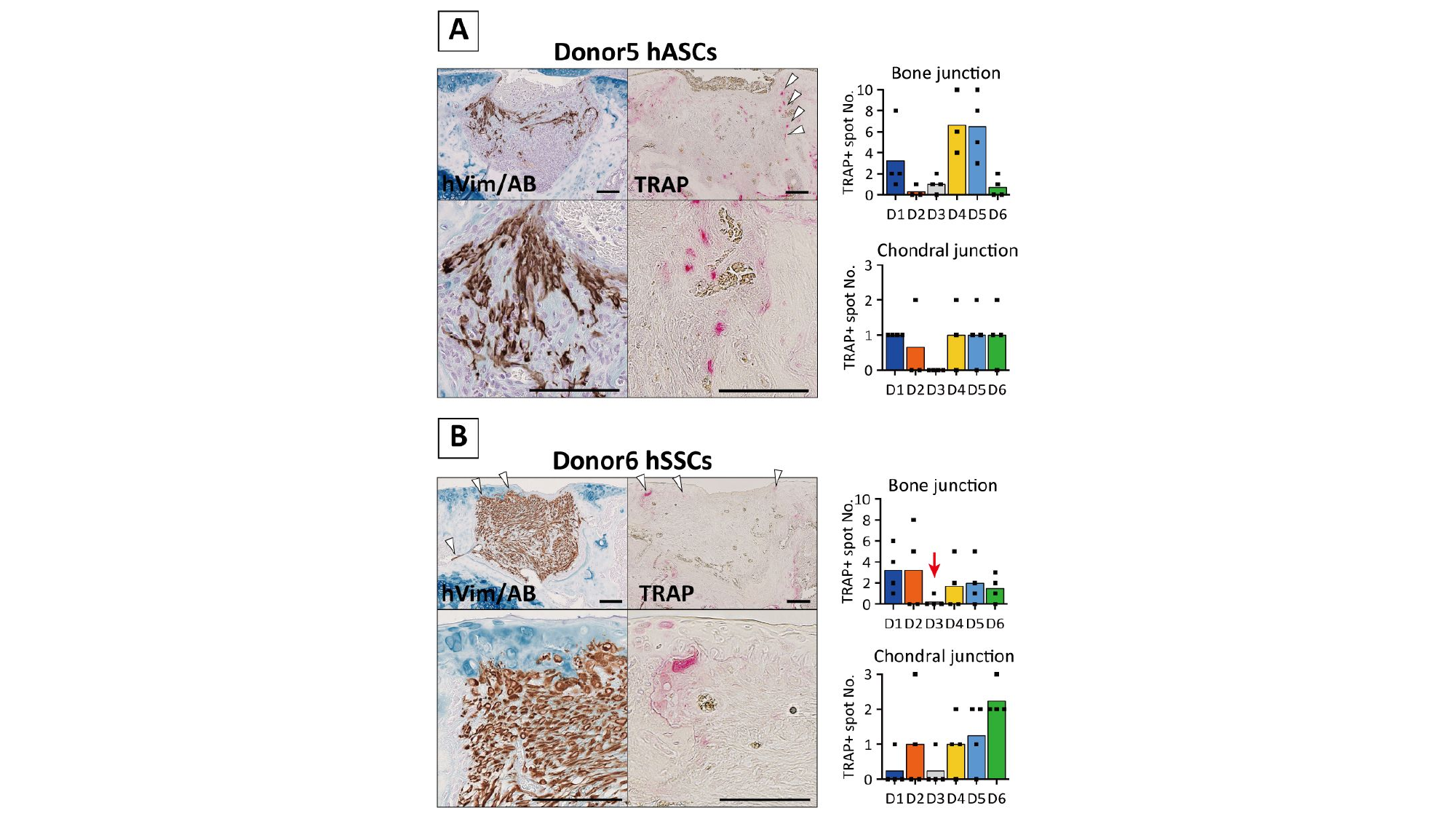

Supplement: Supplementary file 21 — Additional file 21. (A, B) Representative images for IHC for hVimentin and TRAP staining from the samples transplanted with hASCs or hSSCs at day 28. Arrow heads indicate bone erosion spots. The number of TRAP positive spot at border between neo tissue and host bone or articular cartilage are expressed as a bar plot with a dot plot. Bars = 100 μm. [file 13287_2021_2485_MOESM21_ESM.pptx]
